# Synthesis of depression outcomes reported on different scales: A comparison of methods for modelling mean differences

**DOI:** 10.1017/rsm.2025.7

**Published:** 2025-03-17

**Authors:** Beatrice C. Downing, Nicky J. Welton, Hugo Pedder, Ifigeneia Mavranezouli, Odette Megnin-Viggars, A.E. Ades

**Affiliations:** 1 Population Health Sciences, Bristol Medical School, University of Bristol, Bristol, UK; 2 Centre for Outcomes Research and Effectiveness, Research Department of Clinical, Educational & Health Psychology, University College London, London, UK

**Keywords:** meta-regression against baseline severity, network meta-analysis, patient and clinician reported outcomes, ratio of means, standardised mean difference

## Abstract

Several methods have been proposed for the synthesis of continuous outcomes reported on different scales, including the Standardised Mean Difference (SMD) and the Ratio of Means (RoM). SMDs can be formed by dividing the study mean treatment effect either by a study-specific (Study-SMD) or a scale-specific (Scale-SMD) standard deviation (SD). We compared the performance of RoM to the different standardisation methods with and without meta-regression (MR) on baseline severity, in a Bayesian network meta-analysis (NMA) of 14 treatments for depression, reported on five different scales. There was substantial between-study variation in the SDs reported on the same scale. Based on the Deviance Information Criterion, RoM was preferred as having better model fit than the SMD models. Model fit for SMD models was not improved with meta-regression. Percentage shrinkage was used as a scale-independent measure with higher % shrinkage indicating lower heterogeneity. Heterogeneity was lowest for RoM (20.5% shrinkage), then Scale-SMD (18.2% shrinkage), and highest for Study-SMD (16.7% shrinkage). Model choice impacted which treatment was estimated to be most effective. However, all models picked out the same three highest-ranked treatments using the GRADE criteria. Alongside other indicators, higher shrinkage of RoM models suggests that treatments for depression act multiplicatively rather than additively. Further research is needed to determine whether these findings extend to Patient- and Clinician-Reported Outcomes used in other application areas. Where treatment effects are additive, we recommend using Scale-SMD for standardisation to avoid the additional heterogeneity introduced by Study-SMD.

## Highlights

### What is already known

1. To synthesise continuous outcomes measured on different scales, mean treatment effects can be standardised by dividing by the study-specific SD or by a scale-specific SD. Alternatively, a Ratio of Means (RoM) approach expresses all treatment effects as a ratio.
2. Standardisation assumes treatments act additively; RoM assumes multiplicative effects.
3. Treatment effects measured on scales that consist of sums of correlated 2-, 3-, or 4-item subscales are expected to act multiplicatively.

### What is new

1. In a network meta-analysis of treatments for depression, heterogeneity (measured by shrinkage) was lowest for RoM, suggesting treatments for depression may act multiplicatively on the commonly used scales.
2. Standardisation by scale-specific SD (Scale-SMD) was superior to standardisation by study-specific SD (Study-SMD), giving better model fit and lower heterogeneity.
3. We suggest five markers of multiplicative treatment effects, four of which are satisfied by depression scores.
4. The choice of scale model had a limited impact on treatment recommendations.

### Potential impact for RSM readers outside the authors’ field

1. Prior to selecting the approach for synthesising continuous outcomes reported on different outcome scales, it is necessary to establish whether interventions act in an additive or multiplicative fashion.
2. For outcome scales that are additive, we recommend standardising by a scale-specific SD, to avoid the additional heterogeneity introduced by study-specific standardisation.

## 1 Introduction

Several methods have been proposed for the synthesis of continuous outcomes measured on different scales.^1^ These can be sorted into two broad groups. In the first group, the mean treatment effect from each trial is “standardised” by dividing it by a constant, with the aim of expressing all treatment effects in the same units. This assumes that the scales are linearly related and that treatment acts in an additive fashion. Methods of this type can be further subdivided, according to whether the dividing constant is trial-specific,^2^ or scale-specific^3^ ^,^ ^4^; this is discussed below. The second approach is the synthesis of the Ratio of Means (RoM).^5^ While standardisation assumes that treatment acts on measurements in an additive way, RoM assumes that treatment acts to multiply or divide scores.

Most tutorial and methodological guidance papers have described multiplicative and additive approaches as alternative modelling options, without recommending one over the other.^4^ ^,^ ^6^ ^–^ ^8^ A study of 232 systematic reviews^9^ failed to show a clear advantage of either method over the other on measures of between-study heterogeneity. This may, however, have been due to the wide variety of continuous outcomes included in that review. It may be expected that the properties of the specific measurement scales determine whether additive or multiplicative approaches are the most appropriate. For example, many biological measurements, such as cell counts and concentrations in body fluids, are frequently transformed to the log-scale prior to statistical analysis, suggesting that multiplicative models are more appropriate. In studies of depression, a positive relation between the treatment effect and baseline severity is frequently reported,^10^ ^–^ ^13^ which could be interpreted as indicating that treatment effects are proportional rather than additive. Moreover, depression scales are sums of correlated 2-, 3-, or 4-category sub-scales, with a zero origin: based on statistical theory they would be expected to be Poisson-distributed, heteroscedastic, and transformed to normality by log transformations. Patient- and Clinician-Reported outcomes (PCROs) of this type contrast with many of the measurement scales used in educational research, where meta-analytic methods originated,^2^ ^,^ ^14^ that have been constructed to be additive on a natural scale.

By far the most common form of standardisation is to divide mean treatment differences by the study-specific standard deviation (SD), to produce the classic Standardised Mean Difference (SMD). Study-based standardisation assumes that all studies using the same scale have the same SD.^15^ However, due to population differences and sampling error, SDs inevitably vary across studies reporting results on the same scale, introducing noise,^6^ and potentially bias if they vary systematically. To avoid this, standardisation based on division by a scale-specific constant, rather than a study-specific constant, has been proposed. One option is to divide mean differences by the Minimal Clinically Important Difference (MID) for each scale.^1^ ^,^ ^4^ ^,^ ^16^ In a similar vein, Hunter and Schmidt (2004) recommended dividing by the SD in a reference population (the reference SD) for that scale, recognising that variation in SD, which they termed “range variation”, would create artefacts that should be removed to reveal the true treatment effects.^17^ Unfortunately, neither MIDs nor reference SDs have been published for the vast majority of outcome scales. A simple workaround,^18^ which we adopt here, is to approximate a reference SD for each scale by taking the average baseline SD over all the trials reporting results for that scale in the evidence synthesis. Alternative SD and MID approaches are described in the Discussion.

To summarise the properties of the scales, we start from the standard model in which mean treatment effects are seen as the sum of a study component 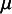 and relative effect 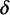 . The hypothesis proposed by Study-SMD is that with mean outcomes 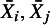 , measured on two scales 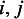 , there are standardizing constants 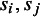 such that differences between the standardised mean outcomes are a constant, independent of treatment:

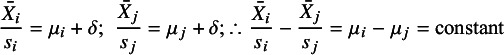



This assumes that the standardising constants are constant between trials, contrary to the facts of range variation. The Scale-SMD hypothesis is exactly the same, except that range variation is eliminated by fixing the standardising constants for each scale.

The RoM hypothesis is that the ratio of the mean outcomes is constant, independent of treatment. 

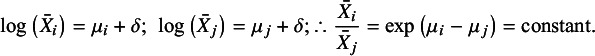



In this paper, we compare the performance of different standardisation and ratio methods in a network meta-analysis of 14 treatments for depression, reported on five different scales. We compare Study-SMD and Scale-SMD, with and without meta-regression (MR) against baseline SD, and RoM. Our objective is to develop methods for deciding which of these models provides the best fit to the data, the most precise estimates, and the lowest between-study heterogeneity. We illustrate these methods by applying them to a frequently used form of PCRO data. We also investigate the impact of model choice on treatment recommendations.

## 2 Methods

### 2.1 Dataset: Pharmacological treatments for depression

**Figure 1:**
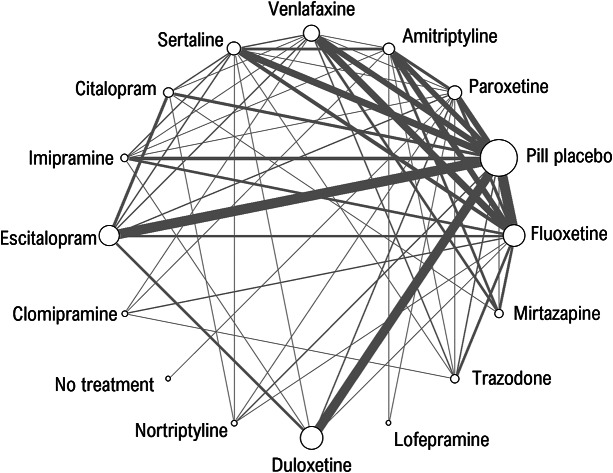
Network of evidence on 14 pharmacological treatments of more severe depression, no treatment and pill placebo. The thickness of edges is proportional to the number of studies and the size of nodes is proportional to the number of participants receiving the treatment.

We use a dataset of 161 studies which were a subset of studies from NICE guideline NG222 that compared pharmacological treatments for more severe (moderate and severe) depression in adults from a larger review.^19^ Studies were included only if they reported findings on the Hamilton Depression Rating Scale (HAMD-17, HAMD-21, HAMD-24), the Beck Depression Inventory (BDI), or the Montgomery-Asberg Depression Scale (MADRS). There were comparisons between 14 active treatments, pill placebo, and “no treatment”. Two studies that reported exceptionally low standard deviations (0.46 and 0.65 on the HAMD-21 scale) were excluded. The evidence network was highly connected with multiple trials of each active treatment (Figure 1). There was direct evidence on 59 of the possible 120 pair-wise comparisons, and they were informed by between 1 and 14 trials (median 2 trials). Because we wished to compare models with and without meta-regression, only studies reporting baseline severity for the whole sample were included.

Data were extracted for NG222 in one of three different formats in the following priority order:

- mean change-from-baseline (CFB) with standard error for each study arm
- mean depression scores at baseline and follow-up with standard errors for each study arm
- mean depression score at follow-up, with standard error for each study arm

following guidance in Guideline Methodology Document 2^18^ for data extraction to fit SMD models.

Network diagrams of trials with data in these three formats, along with the types of data (by format and scale) extracted for each treatment, are shown in the Supplementary Materials (Supplementary Figure S1 and Supplementary Table S1, respectively). However, when fitting a RoM model, the recommended^18^ priority order of data extraction would instead be formats (ii), (iii), then (i), since an assumption of additivity is inherent in calculating CFB, and therefore it is least preferred when fitting a multiplicative model. We, therefore, ran a sensitivity analysis using this alternative prioritisation of data formats; however, this was constrained by available data in the NG222 dataset, as we did not re-extract data from these studies.

For studies reporting baseline and follow-up measures, we need to assume a value for the correlation, 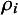 , between baseline and follow-up outcomes to form the standard error of the CFB, and also to estimate RoM from studies that only report in CFB data format (see Supplementary Materials, Appendix 1). The correlation was assumed to be 0.3, which was the median correlation in the studies where it could be estimated in the NG222.^19^ As a sensitivity analysis, we also examined results with the correlation set to 0.5.

### 2.2 Descriptive analyses

Two descriptive analyses were run, to test or to assess the assumptions of the different models. Bartlett’s test of equality of variances was applied to assess the assumption of equality of SDs in different studies reported on the same scale.^20^ The relation between mean scores at baseline (or follow-up) and the SD of scores at baseline (or follow-up) is an indicator of heteroscedasticity.

### 2.3 Standardised Mean Difference (SMD) measures

The standardised mean difference divides the mean difference between treatment arms by a standardising constant 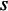 .

For our depression model, the mean difference represents the difference between treatments in the mean CFB. Let 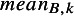 be the mean outcome at baseline and 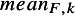 the mean outcome at follow-up for treatment 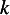 , then the SMD for the mean CFB is:
(1)

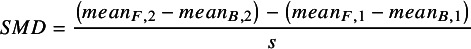



We prioritise modelling the CFB when possible, as it accounts for baseline differences, but we can use follow-up means if no other data are available.

We explore two alternative standardising constants 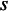 , one using the study-specific SD and the other using a scale-specific SD, and refer to the resulting SMD as Study-SMD and Scale-SMD respectively.

#### 2.3.1 Study-specific SMD

The most common approach is to standardise by the pooled baseline SD specific to the study, termed Cohen’s *d.*^21^ In this case, the SDs at baseline from each arm, 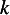 , are combined to give a single pooled SD for the study 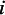 :
(2)

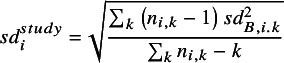

 where 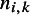 is the number of patients randomised, and 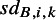 is the baseline SD for study 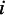 arm 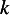 . We use the baseline SD to compute the standardising constant because it is not influenced by treatment, so better reflects the SD of the outcome scale in the population recruited to the study and can be estimated from all trial arms. Note, that we do not consider the situation where interest is in the treatment effect on standard deviation of outcomes.

#### 2.3.2 Scale-specific SMD

Ideally, a scale-specific standardisation constant would be obtained from a large representative population where all scales have been measured. However, in the absence of such a study, we can pool the study-specific baseline SDs, 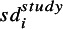 , across all those studies that report on a specific scale 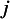 :
(3)

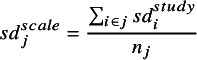

 where 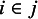 indicates studies reporting scale 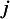 , and 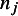 the number of studies that report scale 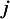 . 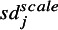 is used as the standardising constant for those studies reporting scale 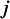 .

### 2.4 Ratio of Means (RoM) measure

If both baseline and follow-up measures are available then a multiplicative effect measure for the ratio change from baseline is the Ratio of Ratio of Means (RoRoM), calculated as:
(4)

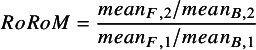



If only follow-up measures are available, then the Ratio of Means (RoM) is:
(5)

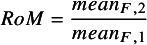



Similarly to CFB, RoRoM adjusts for baseline imbalances, and so is preferred over RoM when it can be calculated. However, both RoRoM and RoM can be pooled to obtain an estimate of the treatment effect.

The RoM is not easily interpretable when outcome scores can have both positive and negative signs. However, if baseline values are available, they can be combined with the CFB to obtain baseline and follow-up means so that the RoRoM in equation (4) can be calculated (see Appendix 1).^18^

### 2.5 Network Meta-Analysis (NMA) model

Network meta-analysis (NMA) enables evidence to be pooled on multiple treatments where the evidence forms a connected network (Figure 1). We used a Bayesian NMA model,^22^ ^,^ ^23^ where we adapted the likelihood and link functions to pool the various data formats to inform the SMD and RoM models (see Supplementary Materials, Appendix 1). The NMA model is put on the parameter 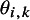 for arm 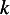 of study 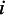 which represents the standardised mean for the SMD models, and represents the log-mean outcome for the ROM model. The NMA model is the same for the SMD and RoM models, but the parameters have different interpretations:
(6)

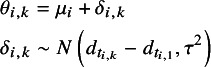

 where 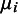 is the standardised mean (or log-mean) for the treatment on arm 1 of study 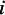 , and 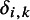 is the study-specific SMD (or log RoM) for arm 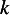 relative to arm 1 of study 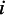 . For a random effects NMA it is assumed that the study-specific SMDs (or log RoMs) come from a Normal distribution, where 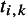 indicates the treatment on arm 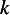 of study 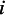 , 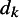 represents the SMD (or log RoM) for treatment 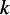 relative to treatment 1 (the reference treatment for the network), and 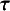 represents the between-study standard deviation on the linear predictor scale. Based on previous analyses of treatments for depression it is expected that there will be a degree of heterogeneity, and so we did not fit fixed effect models.

Estimates of the pooled RoM for treatment 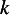 relative to treatment 1 is obtained by exponentiating the log RoM:
(7)

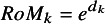



By using the exact same dataset and likelihood for all the models, we are able to combine multiple data types (follow-up scores and CFB) and to provide valid comparisons of goodness of fit statistics for all five models.

### 2.6 Meta-regression of SMD on baseline severity

Versions of both Scale- and Study-SMD models were created in which a regression term for baseline severity was introduced in all active vs inactive comparisons, which we term Scale-SMD-MR and Study-SMD-MR respectively (see Appendix 1 for details).

### 2.7 Model comparison and selection

The models we compared were: SMD standardised with study-specific SD (Study-SMD); SMD standardised with scale-specific SD (Scale-SMD); Study-SMD with meta-regression for severity (Study-SMD-MR); Scale-SMD with meta-regression for severity (Scale-SMD-MR); and RoM. Models were compared using the posterior mean residual deviance, 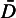 , as a measure of model fit, and the Deviance Information Criterion (DIC), which penalises the deviance by a measure of model complexity, the number of effective parameters, 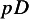 .^24^ 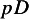 is calculated as the sum over study arms of the difference between the posterior mean deviance and the deviance evaluated at the posterior mean of the mean value of 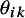 . Models with lower 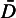 and DIC are preferred.

We report the posterior median and 95% credible intervals for the between-study standard deviation, as a measure of heterogeneity. However, it is important to note that these are not comparable across different outcome scales. To overcome this, we introduce a scale-independent measure of heterogeneity, percentage shrinkage, that can be calculated using 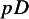 .

For a fixed effect model, all study estimates are equal for the same comparison, so there is no heterogeneity ( 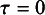 ), 100% shrinkage and the effective number of model parameters is 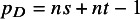 , for the 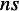 study baseline parameters, and 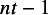 treatment effects relative to reference treatment 1. At the other extreme, for an “independent effects” model where each study effect is independent of all other study effects, there is a high value of 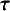 , which will depend on the scale of analysis, 0% shrinkage, and the effective number of model parameters is 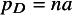 , for the number of study arms 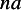 , reflecting a different parameter for each study arm, which is an upper bound for 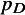 . Random effects models with a value of 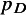 lying between these extremes exhibit a degree of heterogeneity, with values closer to the upper bound indicating higher levels of heterogeneity. We measure this using the % shrinkage defined as:
(8)

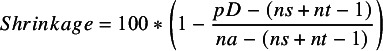

 with low heterogeneity having a % shrinkage closer to 100%, and those with higher heterogeneity having a % shrinkage closer to 0%.

Where baseline and CFB or baseline and follow-up values are used in a bivariate likelihood, there are two data points per study arm, so 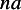 is replaced with 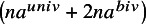 in (9), where 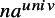 is the number of univariate study arms and 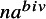 is the number of bivariate study arms.

In the depression example, 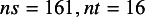 , and 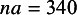 , with 271 study arms reported as CFB or baseline and follow-up, 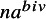 , and 69 study arms reported as final values, 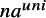 , so 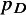 lies between 176 and 611.

### 2.8 Transformation of SMDs and RoM to a common measurement scale (HAMD-17)

To compare treatment effects from SMD and RoM models, the relative treatment effects 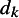 were transformed to a mean difference on the most frequently reported scale: HAMD-17. This required an assumption about the mean and SD on the reference treatment 1 on the HAMD-17 scale. Treatment effects estimated from SMD models, 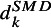 , were back-transformed using the mean pooled baseline SD for the HAMD-17 scale, 
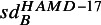
 :
(9)

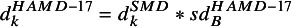



Similarly, between-study SD for the SMD models was back-transformed onto the HAMD-17 scale using the mean pooled baseline SD for the HAMD-17 scale, 
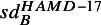
 .

Treatment effects estimated from the RoM model were back-transformed to a mean difference on the HAMD-17 using a representative mean HAMD-17 score on the reference treatment, 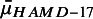 :
(10)

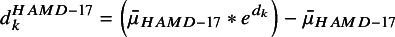

 where 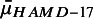 was the CFB calculated from the mean at follow-up values (−6.4 and 15.7 respectively) taken from the largest study reporting depression scores for pill placebo (the reference treatment) on the HAMD-17 scale.^25^ ^,^ ^26^

### 2.9 Treatment recommendations

We also compare the impact of the different models on resulting treatment recommendations based on five different decision rules:

- the treatment with the highest posterior mean estimate of efficacy.
- all treatments where the 95% credible interval (CrI) of the treatment effect relative to pill placebo did not include zero.
- all treatments where the 95% CrI of the treatment effect relative to pill placebo was greater than a clinically important difference of 1 unit on the HAMD-17 scale, which is approximately 0.25 SD units (Table 1).
- As (iii) above, but limited to treatments that were not inferior to the best treatment (ie the 95%CrI for the difference did not include zero).
- The set of treatments that satisfy decision rule (iii) and which were not inferior to any other treatment (ie the 95% credible interval on the difference does not include 0). This rule is a version of a proposal from the GRADE Working Party, with the threshold value set to zero.^27^

**Table 1:** Variation in baseline SD within each scale and regression of baseline SD against baseline severity

Scale	N studies (participants)	Pooled SD at baseline		Difference max–min score	Ratio max:min pooled SD	Bartlett test	Slope: baseline SD vs baseline severity
				Mean	Range		
HAMD–17	78 (150,90)	4.01	1.55, 7.22	52	4.7	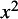 =1725, df = 77, p < 0.0001	0.08 (−0.01, 0.18)
HAMD–21	25 (3,807)	4.63	1.78, 6.40	52	3.6	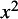 =437, df = 24, p < 0.0001	0.15 (0.01, 0.29)
HAMD–24	5 (865)	4.75	3.36, 5.65	76	1.7	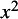 =26.0, df = 4, p < 0.0001	0.16 (−0.39, 0.72)
MADRS	52 (11,627)	5.15	3.35, 10.56	60	3.2	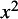 =1413, df = 51, p < 0.0001	−0.17 (−0.30, −0.04)
BDI-I	1 (97)	6.37	–	63	-	*Not estimable*	*Not estimable*

### 2.10 Software, model estimation, and priors

Models were estimated by Bayesian Markov chain Monte Carlo in WinBUGS 1.4.3.^28^ Models were run with three chains with the first 40,000 iterations discarded as burn-in. Chain convergence was assessed with Brooks-Gelman-Rubin plots and chain mixing with trace and history plots. Following burn-in and convergence, 80,000 iterations from each of the three chains were used for inference.

Vague priors were provided for estimates of treatment effect, 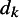 , study-level baselines, 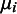 , and between-study SD, 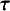 .

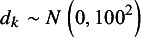




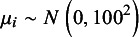



(11)

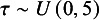



Data visualisation was performed in R version 4.3.1^27^ using packages ggplot2, ggpubr, and viridis.

## 3 Results

### 3.1 Descriptive analysis

Bartlett tests^20^ of the null hypothesis of equal variance in studies reporting on the same scale indicated statistically large differences between the baseline standard deviations (SDs) from each study. The ratio of maximum to minimum baseline SDs varied from 1.7 (HAMD-24) to 4.7 (HAMD-17) (Table 1), with a weighted average of 4.0.

**Figure 2:**
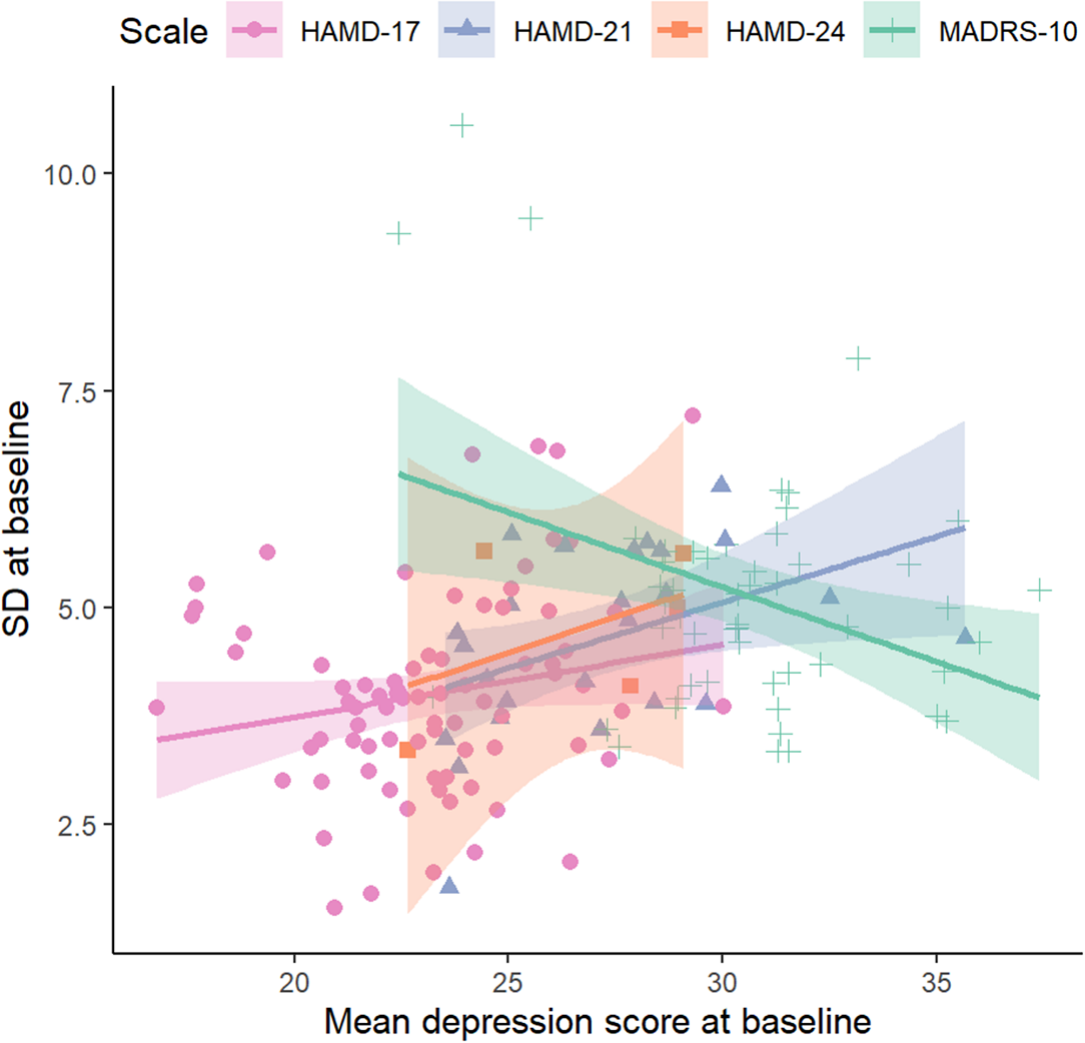
The relationship between the baseline pooled standard deviation and mean depression score at baseline. Results are shown by scale for HAMD-17, HAMD-21, HAMD-24 and MADRS scales. BDI-I could not be plotted because it was reported in a single study.

For the three HAMD scales, mean depression scores at baseline increased with baseline SD, but this was not observed with the MADRS scale (Table 1, Figure 2).

**Table 2:** Model fit statistics and heterogeneity estimated within each of the five models

Model	Dbar	pD	DIC	Regression coefficient mean (95% CrI)	% shrinkage	Between-study SD Scale: linear predictor Median (95% CrI)	Between-study SD Scale: HAMD-17 Median (95% CrI)
Study SMD	625.3	538.2	1163.6	–	16.7	0.34 (0.27, 0.41)	1.35 (1.10, 1.65)
Study SMD (MR)	625.3	537.5	1162.8	−0.168 (−0.333, −0.002)	17.0	0.33 (0.27, 0.40)	1.33 (1.08, 1.62)
Scale SMD	630.6	531.7	1162.3	–	18.2	0.29 (0.23, 0.36)	1.18 (0.93, 1.45)
Scale SMD (MR)	631.3	531.7	1163.0	−0.121 (−0.272, 0.030)	18.3	0.29 (0.23, 0.36)	1.17 (0.92, 1.44)
Ratio of means	629.0	521.9	1150.9	–	20.5	0.09 (0.07, 0.12)	Not calculable

*Note*: Fit statistics: posterior mean residual deviance (relative to 611 data points), DIC, and percentage shrinkage. Coefficient of meta-regression (MR) on baseline severity. The posterior median of between-study SD with the 95% credible interval (CrI) on both the linear predictor scale and, for SMD models, on the HAMD-17 scale.

### 3.2 Model fit, heterogeneity, relative effects, and their precision

Study-SMD and Study-SMD-MR models have a slightly lower posterior mean residual deviance (Table 2), indicating a better fit compared to the other measures. The closer fit is the result of greater model complexity, reflected in the higher 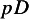 , and larger heterogeneity in treatment effects, as seen in the between-study SD. RoM has the best fit given model complexity with the lowest DIC. Scale-SMD and Scale-SMD-MR models have a similar fit to RoM, but higher DIC reflecting the higher effective number of parameters. These results on DIC reflect the greater shrinkage (reduced heterogeneity) seen with RoM (20.5%), with an intermediate degree of shrinkage with the Scale-SMD (18.2%), and lower shrinkage (higher heterogeneity) with Study-SMD (16.7%). The degree of heterogeneity, as measured by the between-study SD, was 13% lower in the Scale-SMD than in the Study-SMD models (Table 2).

The regression coefficients in the meta-regression models were negative (Table 2), indicating a stronger treatment effect in trials whose study populations were more severely depressed at the outset. This effect was somewhat stronger with the Study-SMD outcome measure. The global fit and shrinkage statistics of the SMD meta-regression models were no different from their non-regression counterparts.

**Figure 3:**
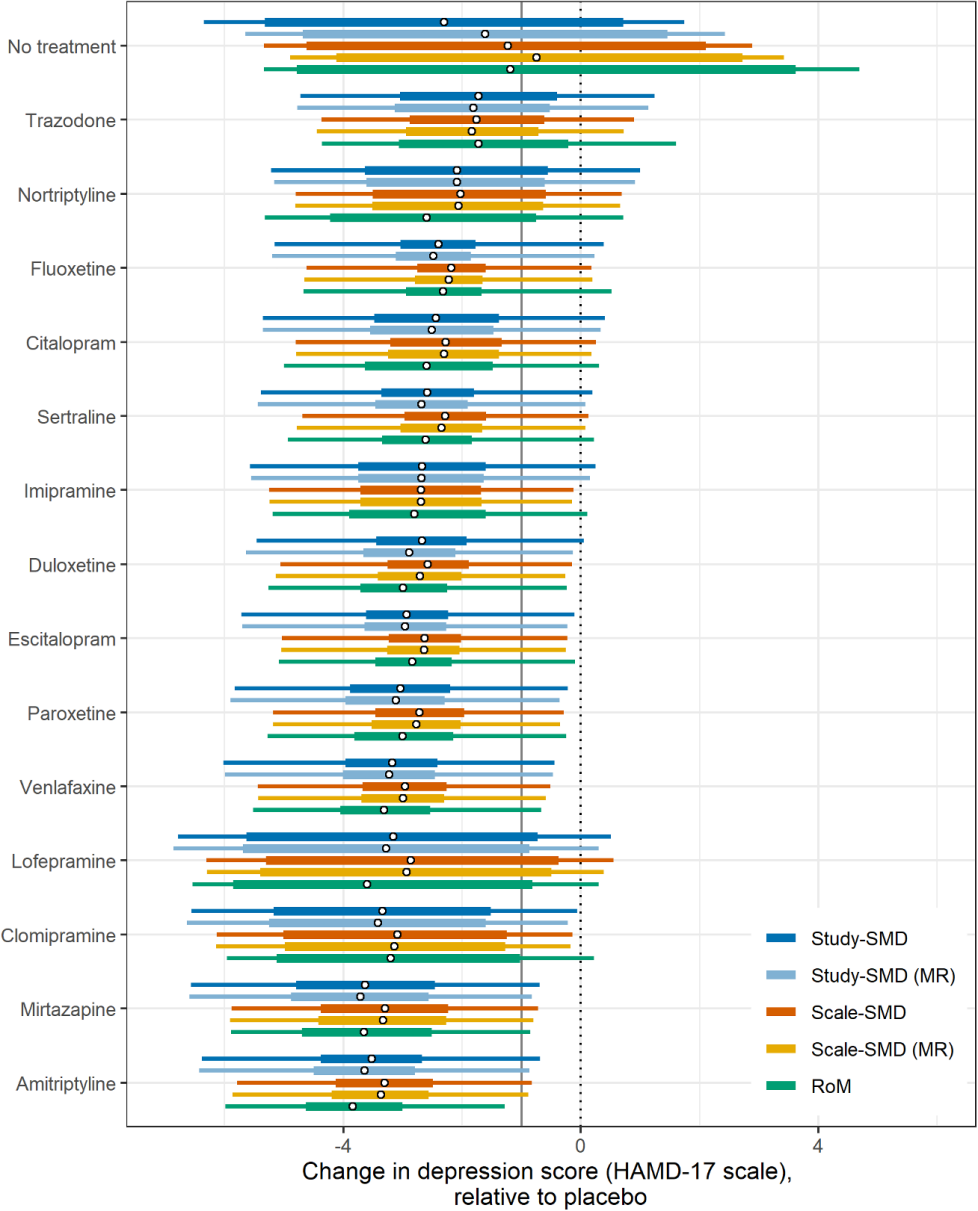
Treatment effect vs placebo as change in depression score on the HAMD-17 scale by model. In each case, circular points indicate the median estimate, thick bars indicate the 95% credible interval (CrI) and thin bars indicate the 95% prediction interval, for each treatment vs placebo. Treatments are ordered by median treatment effect under the RoM model. The vertical grey line indicates one unit on the HAMD-17 scale.

The effects of all treatments relative to pill placebo on the HAMD-17 scale appear in Table 3 and in Figure 3 in the form of a forest plot as median estimates with credible and predictive intervals. Predictive intervals depict where the treatment effect from a new study might lie and are generated from the between-study SD, therefore they reflect the heterogeneity within the modelled data. The predictive intervals were narrower in the Scale-SMD models than in the Study-SMD models.

Effects of active treatments relative to the best treatment on each scale appear in Supplementary Table S2.

### 3.3 Treatment recommendations

The treatment recommendations generated by the different models are presented in Table 4, using five different decision rules. The single best treatment (highest expected efficacy) was Amitriptyline for the RoM model and Scale-SMD models, and Mirtazapine for the Study-SMD models. The second decision rule selected all treatments with evidence of effect “significantly” better than pill placebo (that is where the 95%CrI did not cross zero). All active treatments qualified on all models.

**Table 3:** The mean difference of treatments relative to pill placebo, presented as units on the HAMD-17 scale, with their 95% CrI

SMD by study SD		SMD by study SD (MR on baseline severity)		SMD by scale SD		SMD by scale SD (MR on baseline severity)		RoM	
Mirtazapine	−3.63 (−4.79, −2.46)	Mirtazapine	−3.71 (−4.87, −2.56)	Amitriptyline	−3.30 (−4.13, −2.49)	Amitriptyline	−3.37 (−4.19, −2.56)	Amitriptyline	−3.83 (−4.63, −3.00)
Amitriptyline	−3.52 (−4.38, −2.67)	Amitriptyline	−3.64 (−4.50, −2.79)	Mirtazapine	−3.29 (−4.38, −2.23)	Mirtazapine	−3.34 (−4.42, −2.27)	Mirtazapine	−3.64 (−4.70, −2.51)
Clomipramine	−3.34 (−5.17, −1.52)	Clomipramine	−3.42 (−5.25, −1.60)	Clomipramine	−3.10 (−5.01, −1.25)	Clomipramine	−3.14 (−4.98, −1.27)	Lofepramine	−3.52 (−5.86, −0.82)
Venlafaxine	−3.18 (−3.96, −2.41)	Lofepramine	−3.28 (−5.69, −0.87)	Venlafaxine	−2.96 (−3.68, −2.26)	Venlafaxine	−2.99 (−3.69, −2.30)	Venlafaxine	−3.30 (−4.05, −2.53)
Lofepramine	−3.16 (−5.63, −0.73)	Venlafaxine	−3.23 (−4.01, −2.46)	Lofepramine	−2.86 (−5.30, −0.38)	Lofepramine	−2.95 (−5.40, −0.49)	Clomipramine	−3.17 (−5.12, −1.03)
Paroxetine	−3.03 (−3.89, −2.20)	Paroxetine	−3.12 (−3.96, −2.30)	Paroxetine	−2.72 (−3.46, −1.97)	Paroxetine	−2.77 (−3.52, −2.03)	Paroxetine	−3.00 (−3.82, −2.15)
Escitalopram	−2.93 (−3.62, −2.23)	Escitalopram	−2.96 (−3.64, −2.27)	Imipramine	−2.69 (−3.71, −1.68)	Duloxetine	−2.71 (−3.42, −2.01)	Duloxetine	−2.99 (−3.71, −2.25)
Imipramine	−2.68 (−3.75, −1.60)	Duloxetine	−2.89 (−3.66, −2.11)	Escitalopram	−2.63 (−3.23, −2.02)	Imipramine	−2.69 (−3.71, −1.67)	Escitalopram	−2.83 (−3.46, −2.17)
Duloxetine	−2.68 (−3.44, −1.93)	Imipramine	−2.69 (−3.74, −1.64)	Duloxetine	−2.58 (−3.25, −1.89)	Escitalopram	−2.65 (−3.26, −2.04)	Imipramine	−2.80 (−3.90, −1.61)
Sertraline	−2.59 (−3.36, −1.81)	Sertraline	−2.68 (−3.46, −1.90)	Sertraline	−2.29 (−2.97, −1.59)	Sertraline	−2.35 (−3.03, −1.67)	Sertraline	−2.61 (−3.34, −1.83)
Citalopram	−2.43 (−3.47, −1.38)	Citalopram	−2.51 (−3.55, −1.47)	Citalopram	−2.28 (−3.21, −1.34)	Citalopram	−2.31 (−3.25, −1.37)	Citalopram	−2.58 (−3.64, −1.48)
Fluoxetine	−2.40 (−3.04, −1.78)	Fluoxetine	−2.48 (−3.11, −1.85)	Fluoxetine	−2.18 (−2.75, −1.60)	Fluoxetine	−2.23 (−2.80, −1.65)	Nortriptyline	−2.57 (−4.22, −0.76)
No treatment	−2.31 (−5.32, 0.72)	Nortriptyline	−2.10 (−3.61, −0.61)	Nortriptyline	−2.03 (−3.50, −0.59)	Nortriptyline	−2.07 (−3.52, −0.64)	Fluoxetine	−2.31 (−2.94, −1.67)
Nortriptyline	−2.09 (−3.63, −0.56)	Trazodone	−1.82 (−3.13, −0.52)	Trazodone	−1.75 (−2.88, −0.62)	Trazodone	−1.83 (−2.94, −0.71)	Trazodone	−1.70 (−3.07, −0.21)
Trazodone	−1.72 (−3.04, −0.40)	No treatment	−1.61 (−4.68, 1.46)	No treatment	−1.23 (−4.62, 2.11)	No treatment	−0.73 (−4.12, 2.72)	No treatment	−1.03 (−4.78, 3.62)

*Note*: Estimates are presented from models of depression score standardised as SMDs by study-level and scale-level pooled SD, with and without meta-regression (MR) on baseline severity, and a model of the ratio of means (RoM).

If decision-makers were to recommend all treatments that were better than placebo by at least 1 HAMD-17 unit, the third decision rule, all five models pick out the same 11 treatments, and exclude trazodone, nortriptyline, and lofepramine.

The fourth approach selects from the above 11 treatments all those that are also not inferior to the best treatment. In this case, RoM picks out only seven treatments besides the best, Scale-SMD models a further eight, and Study-SMD models a further nine. Our fifth decision rule, based on GRADE recommendations, identifies a set of treatments that is inferior to no other treatment, again using a 95% credible limit. In this case, all five models picked out the same three treatments. Unlike the first three decision rules, which are driven by differences between active treatments and placebo, the fourth and fifth depend on differences between the active treatments. It seems that discrimination between active treatments is better in RoM models than in Scale-SMD, and that Scale-SMD models discriminate better than Study-SMD.

### 3.4 Sensitivity analyses

The results from the sensitivity analysis using a different prioritisation of data format are given in Supplementary Materials, Appendix 3. The findings were generally similar (Supplementary Materials, Appendix 3, Supplementary Table S3) to those from the main analysis (Table 2), although the benefits of the RoM model compared to the Scale-SMD models were minimal. All five models selected the same treatment as in the main analysis, and the results with the different decision rules differed only slightly from the main analysis (Supplementary Materials Appendix 3). Better discrimination between active treatments on the HAMD-17 scale was observed for the RoM model in both analyses.

A sensitivity analysis setting the before-after correlation at 0.5, as opposed to our base-case 0.3, had negligible effects on shrinkage. Shrinkage was lowered from 17% to 16% for Study-SMD, from 18% to 17% for Scale-SMD, and remained at 21% for RoM.

**Table 4:** Treatment recommendations based on each model, ranked by efficacy, according to five decision rules

Decision rule	Study SMD	Study SMD Meta-regression	Scale-SMD	Scale-SMD Meta-regression	RoM
1. Single best treatment	Mirtazapine	Mirtazapine	Amitriptyline	Amitriptyline	Amitriptyline
2. Treatments better than placebo^a^	All active treatments	All active treatments	All active treatments	All active treatments	All active treatments
3. Treatments better than placebo by 1 HAMD–17^b^	Mirtazapine Amitriptyline Clomipramine Venlafaxine Paroxetine Escitalopram Imipramine Duloxetine Sertraline Citalopram Fluoxetine	Mirtazapine Amitriptyline Clomipramine Venlafaxine Paroxetine Escitalopram Duloxetine Imipramine Sertraline Citalopram Fluoxetine	Amitriptyline Mirtazapine Clomipramine Venlafaxine Paroxetine Imipramine Escitalopram Duloxetine Sertraline Citalopram Fluoxetine	Amitriptyline Mirtazapine Clomipramine Venlafaxine Paroxetine Duloxetine Imipramine Escitalopram Sertraline Citalopram Fluoxetine	Amitriptyline Mirtazapine Venlafaxine Clomipramine Paroxetine Duloxetine Escitalopram Imipramine Sertraline Citalopram Fluoxetine
4. Treatments better than placebo by 1 HAMD–17,^b^ AND no worse than the best treatment^c^	Amitriptyline Clomipramine Venlafaxine Paroxetine Escitalopram Imipramine Duloxetine Sertraline Citalopram	Amitriptyline Clomipramine Venlafaxine Paroxetine Escitalopram Duloxetine Imipramine Sertraline Citalopram	Mirtazapine Clomipramine Venlafaxine Imipramine Paroxetine Escitalopram Duloxetine Citalopram	Mirtazapine Clomipramine Venlafaxine Imipramine Duloxetine Paroxetine Escitalopram Citalopram	Mirtazapine Venlafaxine Clomipramine Duloxetine Paroxetine Imipramine Citalopram
5. GRADE, with a threshold zero. Treatments that are superior to placebo by 1 HAMD–17^b^ and inferior to no other treatment. (Number of treatments satisfying criterion (3) that they were superior to)	Amitriptyline (2) Mirtazapine (2) Venlafaxine (2)	Amitriptyline (3) Mirtazapine (2) Venlafaxine (1)	Amitriptyline (3) Mirtazapine (2) Venlafaxine (2)	Amitriptyline (3) Mirtazapine (2) Venlafaxine (2)	Amitriptyline (4) Mirtazapine (2) Venlafaxine (2)

a X better than Y means that the 95%CrI on the (X–Y) difference did not include zero.

b X better than Y by more than 1 HAMD-17 unit means that the 95% CrI on the (X–Y) difference did not include −1.0.

c X no worse than Y means that the 95%CrI on the (X-Y) difference did not include zero.

## 4 Discussion

### 4.1 Summary of findings

In this dataset, which was typical of pharmaceutical trials for more severe depression, the RoM model performed better than any SMD model. This assessment was based on lower DIC and greater shrinkage of estimates. Furthermore, examination of differences between active treatments’ effects on the HAMD-17 scale produced evidence that treatment effects were estimated with greater precision in the RoM models than in the Scale-SMD model. The Scale-SMD model gave a similar fit to RoM but had higher heterogeneity.

Scale-SMD models performed better than Study-SMD, giving lower heterogeneity and more precise estimates, by about 6%. A core assumption of the Study-SMD method is that all the studies that report on the same scale should have the same SD.^15^ Hunter and Schmidt^17^ regarded the changes in SMDs due to different study variances as “range variation” artefacts that required removing, and they advocated a form of Scale-SMD approach. In our dataset, range variation, which is generated by sampling variation as well as between-study population differences, was statistically and materially highly significant, with study SDs reported on the same scale varying over an average 4.0-fold range. The effect of the Study-SD method is therefore to multiply relative treatment effects by an arbitrary number between 1 and 4. Similar findings have been reported in trials reporting on the Liebowitz Social Anxiety Scale.^29^

Scale-SMD models represent an improvement on Study-SMDs because they avoid range variation by using a single normalising SD for each scale. To create a reference SD we used the average SD over the available studies using that scale. This is an imperfect solution, as it risks introducing random error when there are small numbers of studies. The ideal would be to use reference SDs from a single large population study. However, large population studies looking at the full range of scales are uncommon. Alternatively, it may be possible to derive reference SDs by modelling large collections of trials or observational studies.

### 4.2 Criteria employed in this study, and robustness of findings

The criteria used here to compare models were: global model fit; percentage shrinkage (a scale-independent measure of heterogeneity); DIC, which is model fit penalised for complexity; and precision of relative effects. These are in line with previous literature on the choice of scale in meta-analysis, which has focused on between-study heterogeneity measured by the Q-statistic,^9^ ^,^ ^30^ ^,^ ^31^ or on both Q-statistics and precision of estimates.^32^ ^,^ ^33^ Another measure that has been used is the percentage of study effects within the 95% confidence interval of the pooled effect.^33^ A study comparing five different effect measures in an NMA of trials reporting binary data used global fit statistics and DIC.^34^

A limitation of reliance on aggregated data is that assumptions have to be made about correlations between baseline and follow-up scores. Our results assumed a correlation of 0.3, based on the evidence available; a sensitivity analysis with a correlation of 0.5 showed a greater advantage of RoM models. A second sensitivity analysis examined the priority order for data extraction. The base-case analysis prioritised (i) CFB, (ii) baseline and follow-up, and (iii) follow-up only. Results with an alternative ordering (ii, iii, and i) produced similar findings although there was no longer any difference between Scale-SMD and RoM methods. However, the better discrimination between treatments observed in the base-case analysis was also evident in this sensitivity analysis. It should be noted that this analysis was limited by the available data (which had been extracted following the priorities for SMDs).

### 4.3 Generality of findings

A limitation of the study is that it is restricted to a single dataset of trials, which includes only pharmaceutical interventions, for a particular severity range—more severe—of a specific disorder, depression. It is therefore unclear how far one can generalise from the present results to other PCROs consisting of correlated sums of sub-scale scores, let alone to other types of continuous outcomes. Although studies comparing RoM with Study-SMD in large collections of pair-wise meta-analyses have reported more heterogeneity in SMD models,^9^ this finding may be due to the poor performance of Study-SMD, and the comparison may yield different results if Scale-SMD were used instead. We would in any case urge caution before adopting any “one-size-fits-all” solution, and recommend that further studies are conducted, along the lines of the present study, using large networks of trials to examine commonly used PCROs. Separate investigations are required in each clinical area of psychiatric, neurologic, and other fields where PCROs are used.

### 4.4 Minimally important difference

Another approach, attracting increasing attention, is to standardise by dividing mean treatment effects in each trial by the MID, generating treatment effects in MID units. Some researchers regard MIDs as more easily interpretable than either SMDs or the units of the original scales.^1^ ^,^ ^3^ ^,^ ^16^ Interestingly, in parallel to SMDs, both scale-specific^1^ ^,^ ^16^ ^,^ ^35^ and study-specific^36^ MIDs have been proposed, the latter for studies using scales for which no MID estimate is available. MIDs have also been expressed as percent improvement, indicating a proportional rather than additive effect, most notably in studies of depression.^37^ ^,^ ^38^ The adoption of a MID approach does not in itself, therefore, contribute directly to the issues addressed in this paper: whether treatment effects are additive or multiplicative, and the choice of scale-specific or study-specific standardisation. Researchers using MID face several additional challenges: multiple ways of constructing and/or presenting the MID,^39^ ^,^ ^40^ the extreme variability of estimates,^41^ ^–^ ^43^ and between-patient variation.^38^ ^,^ ^44^ Before choosing a solution to the problems posed by outcomes reported on multiple scales, we believe the first priority must be to determine whether treatment effects are multiplicative or additive.

### 4.5 Impact of choice of scale on treatment recommendations

Although there were differences between the five models in treatment rankings, including differences regarding which treatment was best, there was a high level of concordance. A decision rule similar to the one proposed by the GRADE Working Group^27^ picked out the same three treatments under all five models. The relative insensitivity of treatment rankings to the choice of scale has been observed in previous NMAs of binomial data.^34^ One should not conclude from this that the choice of scale does not matter, as it can have a substantial impact on the estimates themselves and their precision.^34^ The formulation of decision rules for recommending treatments following an NMA is a topic calling for further research, particularly decision rules that take uncertainty into account.^45^ Rules such as those proposed by GRADE,^27^ because they can recommend treatments that are not “significantly” different from the best treatment, may have the unwanted effect of privileging treatments with more uncertain evidence.

### 4.6 Recommendations regarding the choice of scale

A decision about whether treatment effects are multiplicative or additive should be based on all the evidence available, including clinical opinion.^30^ Table 5 summarises the kinds of evidence that would support the conclusion that effects are multiplicative. Depression scales score on the first four of the five criteria. Thus, although the empirical evidence on model fit and shrinkage in this paper is not strong, in combination with other evidence, we would regard the overall evidence for multiplicative effects as strong enough to favour RoM as the default.

**Table 5:** Criteria supporting the assumption of multiplicative treatment effects

	Criteria
1	Superior fit, greater shrinkage, and lower heterogeneity in models with a log link compared to an identity link
2	Increasing SD with increasing baseline severity (heteroscedasticity)
3	Scale constructed from the sum of correlated Poisson variables
4	Individual patient data shows that the treatment effect increases with baseline severity
5	Scores commonly reported and analysed following a log transformation

For measurement scales where additivity can be assumed, we would recommend scale-specific standardisation by SMD or, if reliable estimates exist, MID. This is supported by the results reported here and is based on well-established arguments in textbooks and tutorial papers.^6^ ^,^ ^15^ ^,^ ^17^ It is interesting to note that measurement scales in educational and psychological research, where meta-analytic methods including SMDs originated,^2^ ^,^ ^14^ were generally constructed to be additive. This may explain the durability of additive models in meta-analysis.

### 4.7 Future research directions

The additive SMD and proportional RoM approaches assume, respectively, uniform linearity or uniform proportionality of treatment effects on the underlying depression scale. Rather than enquiring into the relation between the measurement scales and the underlying severity of depression, we should instead consider the relationships between the scales themselves. For this, we need to turn to methods for test equating and linking.^46^ ^,^ ^47^ While proportionality appears to be closer to the truth for the depression scales studied here, based on the Table 5 criteria, test-linking studies show that the assumption of any simple uniform relation can only be an approximation. Studies using three distinct methodologies, factor analysis,^48^ Item Response Theory,^49^ ^–^ ^52^ and equi-percentile linking,^53^ have all shown that each scale’s ability to discriminate different degrees of depression varies unevenly across the severity spectrum and that each scale has a unique sensitivity profile. This is confirmed by recent work on the MID of depression scales, showing that while MID generally increases with baseline severity, the relationship is uneven and scale-dependent.^38^

Further research is required to find ways of leveraging the one-to-one mapping information generated by test-linking studies, to drive algorithms that map between aggregated results (mean and SD) reported on different scales.^54^ Methods of this type would be considerably more flexible than either RoM or SMD, as they would allow synthesis even when scales are not monotonically related.

## Supporting information

Downing et al. supplementary material Downing et al. supplementary material

## Data Availability

The data and code files that support the findings of this study are available from OSF: https://osf.io/2jyf7. The WinBUGS code is also included in the Supplementary Materials (Appendix 2).

## Supplementary material

To view supplementary material for this article, please visit http://doi.org/10.1017/rsm.2025.7.
