# Supplementary material for: Synthesis of depression outcomes reported on different scales: A comparison of methods for modelling mean differences
Source: Res Synth Methods. 2025 Mar 17;16(3):460–78. doi: 10.1017/rsm.2025.7 (PMC12527519; doi:10.1017/rsm.2025.7)
Supplement: Downing et al. supplementary material [file S1759287925000079sup001.docx]

# **Supplementary Materials**

**Synthesis of depression outcomes reported on different scales: a comparison of methods for modelling mean differences.**

Downing BC^1^*, Welton NJ^1^, Pedder H^1^, Mavranezouli I^2^, Megnin-Viggars O^2^, Ades AE^1^

1 – Population Health Sciences, Bristol Medical School, University of Bristol, Whatley Road, Bristol, BS8 2PS, UK

2 – Centre for Outcomes Research and Effectiveness, Research Department of Clinical, Educational & Health Psychology, University College London, London, UK

*beatrice.downing@bristol.ac.uk

**Figure S1**

**Tables S1-S2**

**Appendix 1: Methodological Details**

**Appendix 2: WinBUGS Code**

**Appendix 3: Sensitivity analysis 1, preference of reporting format**

**Appendix 4: Sensitivity analysis 2, correlation between baseline and follow-up scores**

**References**

Five scales SUPP revised 2024.10.22

**
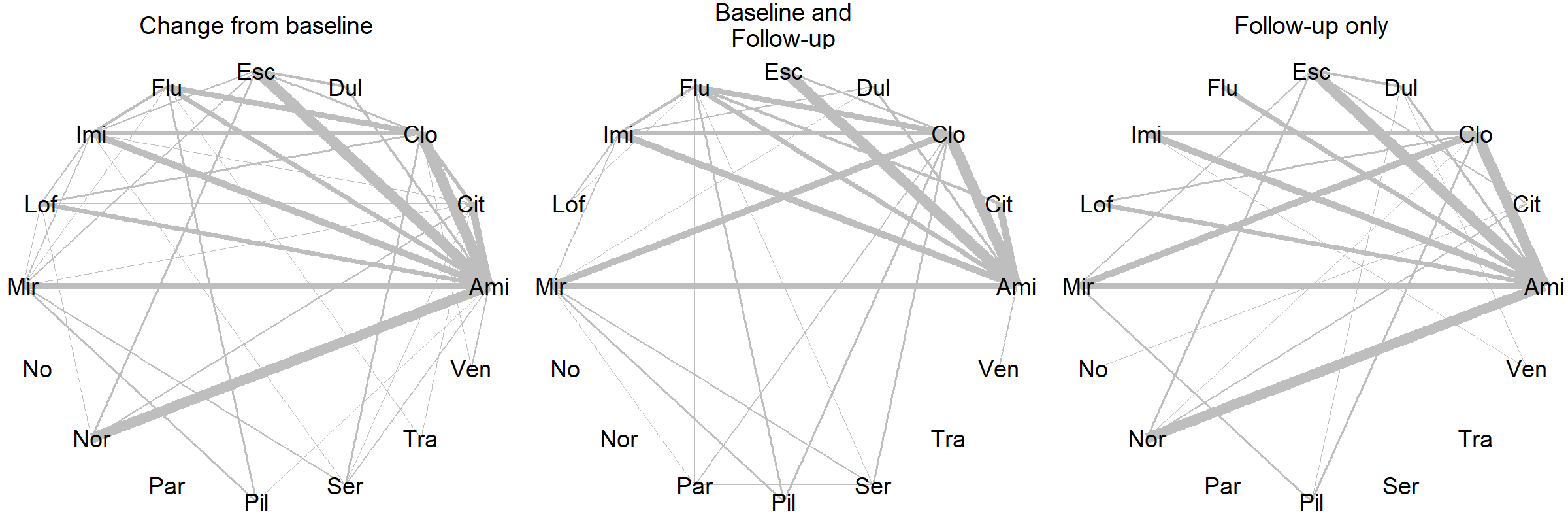
**

**Figure S1.** Networks of evidence shown by data type extracted: change-from baseline scores, baseline and follow-up scores and follow-up score only.

**Table S1**. Summary of evidence for each treatment: number of study arms by data type and scale.

| Treatment | Study arms | Study participants |  | Number of study arms by data type | | |  | Number of study arms by scale | | | | |
| --- | --- | --- | --- | --- | --- | --- | --- | --- | --- | --- | --- | --- |
|  |  |  |  | CFB | Baseline and  follow-up | Follow-up |  | BDI-I | HAMD-17 | HAMD-21 | HAMD-24 | MADRS |
| All | 340 | 31484 |  | 169 | 102 | 69 |  | 2 | 166 | 53 | 10 | 109 |
|  |  |  |  |  |  |  |  |  |  |  |  |  |
| Amitriptyline | 30 | 1576 |  | 10 | 18 | 2 |  | 0 | 17 | 11 | 0 | 2 |
| Citalopram | 12 | 1251 |  | 4 | 4 | 4 |  | 0 | 3 | 0 | 0 | 9 |
| Clomipramine | 4 | 192 |  | 0 | 4 | 0 |  | 0 | 2 | 0 | 0 | 2 |
| Duloxetine | 23 | 4173 |  | 15 | 1 | 7 |  | 0 | 15 | 1 | 2 | 5 |
| Escitalopram | 29 | 3572 |  | 16 | 5 | 8 |  | 0 | 8 | 0 | 0 | 21 |
| Fluoxetine | 50 | 3828 |  | 20 | 21 | 9 |  | 1 | 26 | 12 | 1 | 10 |
| Imipramine | 12 | 742 |  | 8 | 2 | 2 |  | 0 | 7 | 2 | 0 | 3 |
| Lofepramine | 2 | 99 |  | 2 | 0 | 0 |  | 0 | 1 | 0 | 0 | 1 |
| Mirtazapine | 11 | 882 |  | 3 | 4 | 4 |  | 0 | 6 | 1 | 0 | 4 |
| Nortriptyline | 6 | 219 |  | 2 | 1 | 3 |  | 1 | 3 | 0 | 2 | 0 |
| Paroxetine | 24 | 2179 |  | 14 | 5 | 5 |  | 0 | 10 | 7 | 0 | 7 |
| Pill placebo | 72 | 7294 |  | 44 | 15 | 13 |  | 0 | 38 | 10 | 4 | 20 |
| Sertraline | 26 | 1983 |  | 12 | 8 | 6 |  | 0 | 14 | 2 | 1 | 9 |
| Trazodone | 10 | 867 |  | 7 | 3 | 0 |  | 0 | 8 | 1 | 0 | 1 |
| Venlafaxine | 28 | 2531 |  | 12 | 11 | 5 |  | 0 | 8 | 6 | 0 | 14 |
| No treatment | 1 | 96 |  | 0 | 0 | 1 |  | 0 | 0 | 0 | 0 | 1 |

**Table S2**. Treatment effect relative to the most effective treatment for the subset of treatments judged to be effective relative to pill placebo (95% CrI for treatment effect not including zero). Treatment differences are mean differences on the HAMD-17 scale, relative to the treatment at the top of the column (that with the largest mean effect relative to pill placebo).

| SMD by study SD | | SMD by study SD  (MR on baseline severity) | | SMD by scale SD | | SMD by scale SD  (MR on baseline severity) | | RoM | | |
| --- | --- | --- | --- | --- | --- | --- | --- | --- | --- | --- |
| ***Relative to Mirtazapine*** | | ***Relative to Mirtazapine*** | | ***Relative to Amitriptyline*** | | ***Relative to Amitriptyline*** | | ***Relative to Amitriptyline*** | | |
| Amitriptyline | 0.11  (-1.11, 1.31) | Amitriptyline | 0.07  (-1.12, 1.26) | Mirtazapine | 0.01  (-1.11, 1.14) | Mirtazapine | 0.03  (-1.08, 1.16) | Mirtazapine | 0.19  (-0.96, 1.37) |
| Clomipramine | 0.29  (-1.76, 2.33) | Clomipramine | 0.29  (-1.74, 2.32) | Clomipramine | 0.20  (-1.73, 2.11) | Clomipramine | 0.23  (-1.63, 2.19) | Lofepramine | 0.24  (-1.92, 2.89) |
| Venlafaxine | 0.45  (-0.73, 1.64) | Lofepramine | 0.43  (-2.14, 3.02) | Venlafaxine | 0.34  (-0.59, 1.27) | Venlafaxine | 0.38  (-0.54, 1.30) | Venlafaxine | 0.53  (-0.45, 1.49) |
| Lofepramine | 0.47  (-2.10, 3.08) | Venlafaxine | 0.49  (-0.68, 1.65) | Lofepramine | 0.43  (-1.96, 2.85) | Lofepramine | 0.42  (-1.94, 2.81) | Clomipramine | 0.63  (-1.34, 2.86) |
| Paroxetine | 0.60  (-0.74, 1.94) | Paroxetine | 0.59  (-0.72, 1.91) | Paroxetine | 0.58  (-0.34, 1.52) | Paroxetine | 0.60  (-0.32, 1.53) | Paroxetine | 0.83  (-0.18, 1.84) |
| Escitalopram | 0.69  (-0.58, 2.00) | Escitalopram | 0.76  (-0.49, 2.02) | Imipramine | 0.61  (-0.61, 1.83) | Duloxetine | 0.65  (-0.36, 1.69) | Duloxetine | 0.85  (-0.22, 1.91) |
| Imipramine | 0.94  (-0.55, 2.45) | Duloxetine | 0.83  (-0.52, 2.17) | Escitalopram | 0.67  (-0.27, 1.61) | Imipramine | 0.68  (-0.53, 1.91) | Escitalopram | 1.00  (0.03, 1.98) |
| Duloxetine | 0.95  (-0.41, 2.31) | Imipramine | 1.03  (-0.46, 2.50) | Duloxetine | 0.72  (-0.30, 1.76) | Escitalopram | 0.72  (-0.22, 1.68) | Imipramine | 1.03  (-0.26, 2.37) |
| Sertraline | 1.04  (-0.25, 2.31) | Sertraline | 1.03  (-0.24, 2.31) | Sertraline | 1.01  (0.12, 1.91) | Sertraline | 1.02  (0.14, 1.94) | Sertraline | 1.23  (0.27, 2.18) |
| Citalopram | 1.19  (-0.22, 2.62) | Citalopram | 1.20  (-0.19, 2.62) | Citalopram | 1.02  (-0.13, 2.21) | Citalopram | 1.06  (-0.10, 2.22) | Nortriptyline | 1.24  (-0.52, 3.17) |
| Fluoxetine | 1.22  (-0.11, 2.35) | Fluoxetine | 1.23  (-0.13, 2.34) | Fluoxetine | 1.13  (0.34, 1.92) | Fluoxetine | 1.14  (0.35, 1.95) | Citalopram | 1.24  (-0.03, 2.55) |
| Nortriptyline | 1.54  (-0.31, 3.35) | Nortriptyline | 1.62  (-0.20, 3.43) | Nortriptyline | 1.27  (-0.31, 2.85) | Nortriptyline | 1.30  (-0.28, 2.85) | Fluoxetine | 1.51  (0.67, 2.36) |
| Trazodone | 1.91  (0.25, 3.57) | Trazodone | 1.90  (0.26, 3.53) | Trazodone | 1.55  (0.27, 2.84) | Trazodone | 1.54  (0.25, 2.83) | Trazodone | 2.12  (0.64, 3.71) |

# **Appendix 1: Methodological details**

## **Notation**

Let $y_{B,i,k}$ and $sd_{B,i,k}$, and $n_{B,i,k}$ be the mean, standard deviation, and sample size at baseline for study $i$, arm $k$, with standard error $se_{B,i,k}=\frac{sd_{B,i,k}}{\sqrt{n_{B,i,k}}}$. Similarly, let $y_{F,i,k}$ and $sd_{F,i,k}$, $n_{F,i,k}$be the mean, standard deviation, and sample size at follow up with standard error $se_{F,i,k}=\frac{sd_{F,i,k}}{\sqrt{n_{F,i,k}}}$. Let the correlation between baseline and follow-up measures be $\rho_{i,k}$. The mean change from baseline is $y_{CFB,i,k}=y_{F,i,k}-y_{B,i,k}$ with standard deviation $sd_{CFB,i,k}=\sqrt{sd_{B,i,k}^{2}+sd_{F,i,k}^{2}-2\rho_{i,k}sd_{B,i,k}sd_{F,i,k}}$ and standard error $se_{CFB,i,k}=\frac{sd_{CFB,i,k}}{\sqrt{n_{F,i,k}}}$. Studies may report all or a subset of CFB, baseline, and follow-up summaries. If all baseline, follow-up and change from baseline summaries are reported then the correlation can be estimated using the above formula.

***Choice of data format***

In practice, when fitting RoM models, baseline and follow-up scores are the preferred data to extract, because they allow the Ratio of Ratio of Means (RoRoM) to be estimated, which adjusts for baseline imbalance. For the same reason, CFB summaries are the preferred data to extract when fitting SMD models. However, for the comparison of the different models we used the same data format for both models where possible to ensure that both SMD and RoM models were fitted to the same data. Where both CFB and baseline and follow-up scores were available, CFB summaries were used for the base-case dataset to which all models were fitted in the main analysis. A sensitivity analysis was conducted where baseline and follow-up data were used instead of CFB summaries when both formats were available (Appendix 3). Again, in this sensitivity analysis all models were fitted to the same dataset.

## **Likelihood and links for different data formats and models**

### **SMD models: studies reporting CFB summaries**

The preferred data format is CFB data, which is given a bivariate Normal likelihood:

The mean CFB, , is linked to the standardised mean CFB, $\theta_{i,k}$, by

where $j_{i}$ the scale used in study $i$. The NMA model is given to the standardised mean CFB, $\theta_{i,k}$, and treatment effects are SMD in CFB.

### **SMD models: studies reporting baseline and follow-up summaries**

If CFB data are not readily available, then CFB can instead be modelled using baseline and follow-up measures for an assumed correlation between baseline and follow-up $\rho_{i,k}$. A bivariate Normal likelihood is used:

The mean at follow-up, , is linked to the standardised mean CFB, $\theta_{i,k}$, and mean at baseline, , by

where $j_{i}$ the scale used in study $i$. The NMA model is given to the standardised mean CFB, $\theta_{i,k}$, and treatment effects are SMD in CFB. The mean at baseline parameters are nuisance parameters and given flat priors.

### **SMD models: studies reporting follow-up summaries**

If CFB data are not available, and CFB can’t be modelled using baseline and follow-up measures, then we model follow-up data using a Normal likelihood:

The mean at follow-up, , is linked to the standardised mean at follow-up, $\theta_{F,i,k}$, by

where $j_{i}$ the scale used in study $i$. The NMA model is given to the standardised mean at follow-up, $\theta_{F,i,k}$ and treatment effects are SMDs at follow-up. Whilst SMDs at follow-up differ from SMDs in CFB, the same NMA model is used to pool both, under the assumption that treatment differences at follow-up are unbiased estimates of treatment differences in CFB. This holds if randomisation is adequate, and samples are sufficiently large to avoid baseline imbalances.

### **RoM models: studies reporting baseline and follow-up summaries**

For RoM models, data reported as baseline and follow-up summaries allow adjustment for baseline imbalance. A bivariate Normal likelihood is used for baseline and follow-up outcomes using equation . The log-mean at follow-up, , is linked to the log RoM at follow-up relative to baseline, $\theta_{i,k}$, and log-mean at baseline, , by

The NMA model is given to the log RoM at follow-up relative to baseline, $\theta_{i,k}$. The resulting treatment effect estimates are ratios of ratios of means at follow-up relative to baseline (RoRoMs). The mean at baseline parameters are nuisance parameters and given flat priors.

### **RoM models: studies reporting follow-up summaries**

The follow-up summaries are given a Normal likelihood as given in equation . The mean at follow-up, , is linked to the log mean at follow-up, $\theta_{i,k}$, by

The NMA model is given to the log mean at follow-up, $\theta_{i,k}$ and treatment effect estimates are ratios of means at follow-up. The same NMA model is used to pool both RoM and RoRoM, under the assumption that treatment effects at follow-up are unbiased estimates of treatment effects in the ratio of means at follow-up relative to baseline. This holds if randomisation is adequate, and samples are sufficiently large to avoid baseline imbalances.

### **RoM models: studies reporting CFB summaries**

Ratio of mean models cannot be fitted to CFB data unless baseline summaries are also available. If they are available then a bivariate Normal likelihood can be used:

and then the log-mean at follow-up, , is linked to the log RoM at follow-up relative to baseline, $\theta_{i,k}$, and log-mean at baseline, , using equation . The NMA model is given to the log RoM at follow-up relative to baseline, $\theta_{i,k}$ and treatment effect estimates are ratios of means at follow-up. The mean at baseline parameters are nuisance parameters and given flat priors.

## **Meta-regression on baseline severity**

For the SMD models we fitted a meta-regression model by adding a regression term for baseline severity as a covariate to the NMA model (equation ):

$$\theta_{i,k}=\mu_{i}+\delta_{i,k}+\left(\beta_{t_{i,k}}-\beta_{t_{i,1}}\right)*\left(x_{i}-\bar{x}\right)$$

where $x_{i}$is the standardised mean outcome at baseline (averaging over study arms), standardised using either study-specific (equation ) or scale-specific (equation ) SD depending on the SMD model used, $\bar{x}$is a weighted average of standardised means at baseline across studies (used to “centre” the regression), and the regression coefficients $\beta_{k}$depend on treatment:

$$\beta_{k}=\left\{\begin{matrix} 0 & \text{inactive treatments} \\ B & \text{active treatments} \end{matrix}\right.$$

so that the effect of baseline severity is modelled when an active treatment is compared with an inactive treatment, but that the effects “cancel out” when active treatments are compared. The regression coefficient $B$ is given a flat Normal prior, as for the $d_{k}$parameters in equation .

***Imputation of missing standard deviation at follow-up***

Where$sd_{F,i,k}$ was missing for studies reported as CFB, $se_{F,i,k}$, which was required in the specification of the covariance matrix for the bivariate likelihood, was imputed from a linear regression of $sd_{F,i,k}$ upon $sd_{B,i,k}$.

# **Appendix 2: WinBUGS Code**

## Study-SMD

#Study-SMD

#Noting that back-transformations of treatment effects (d)

#and between-study SD (sd) require the mean pooled SD for the HAMD-17 scale.

Model{ # *** PROGRAM STARTS

### SECTION 1 - specifications and transformations, length=1

d[1] <- 0 ## treatment effect is zero for reference treatment

dPred[1] <- 0 ## treatment effect is zero for reference treatment

tau <- pow(sd,-2) ## between-trial precision = (1/between-trial variance)

T[1] <- A[2] ## A[2] is mean follow-up score on HAMD-17 scale, reference placebo arm

baselineSD <- pooledSD_scale[2] ## Pooled SD for baseline (HAMD-17)

sdNat <- sd * baselineSD ## Between-study SD on the natural scale rather than SD scale

totresdev <- sum(resdev[]) ## Total Residual Deviance

### SECTION 2 - priors

sd ~ dunif(0,5) # vague prior for between-trial SD

for (k in 2:nt){ d[k] ~ dnorm(0,.0001) ## vague priors for treatment effects

}

for (i in 1:ns){ mu[i] ~ dnorm(0, 0.001) ## vague priors for study-level means

for (k in 1:na[i]){ phiB[i,k] ~ dnorm(0, 0.0001) ## vague priors for baselines

}

}

### SECTION 3 - treatment effects and transformations

# Calculate absolute differences from relative effects, length=nt-1

for (k in 2:nt){

T[k] <- A[2] + dNatAll[1,k] ## Mean at follow-up on chosen scale at mean severity

MD[k] <- T[k] - T[1] ## treatment effect as mean difference on chosen scale

RoM[k] <- T[k] / T[1] ## treatment effect as ratio on chosen scale

dPred[k] ~ dnorm(d[k], tau)

}

# Calculate all relative treatment differences, array [nt-1, nt]

for (c in 1:(nt-1)) {

for (k in (c+1):nt) {

dAll[c,k] <- d[k] - d[c]

dNatAll[c,k] <- baselineSD*(dAll[c,k])

dPredAll[c,k] <- dPred[k] - dPred[c]

dPredNatAll[c,k] <- baselineSD*(dPredAll[c,k])

}

}

# Ranking on relative scale, length=nt

for (k in 1:nt) {

rk[k] <- rank(d[],k) ## assumes events are "bad" / negative d values are good

best[k] <- equals(rk[k],1) ## probability that treat k is best

for (h in 1:nt){ prob[h,k] <- equals(rk[k],h) } ## probability that treat k is h-th best

}

### SECTION 4 - network meta-analysis model, length = ns (with arm loops within)

for(i in 1:ns){ # LOOP THROUGH STUDIES

w[i,1] <- 0 ## adjustment for multi-arm trials is zero for control arm

delta[i,1] <- 0 ## treatment effect is zero for control arm

resdev[i] <- sum(dev[i,1:na[i]]) ## summed residual deviance for study i

for(k in 1:na[i]) { ## LOOP THROUGH ARMS

## model for linear predictor

theta[i,k] <- mu[i] + delta[i,k]

} ## CLOSE ARM LOOP

for (k in 2:na[i]) { ## LOOP THROUGH ARMS >=2

## treatment difference in study i, arm k

delta[i,k] ~ dnorm(md[i,k],taud[i,k])

## mean and precision of distributions (with multi-arm trial correction)

md[i,k] <- d[t[i,k]] - d[t[i,1]] + sw[i,k]

taud[i,k] <- tau *2*(k-1)/k

## adjustment and cumulative adjustment for multi-arm RCTs

w[i,k] <- (delta[i,k] - d[t[i,k]] + d[t[i,1]])

sw[i,k] <- sum(w[i,1:k-1])/(k-1)

} # END ARM LOOP >=2

} # END STUDY LOOP

### SECTION 5 - estimation specific to studies reporting CFB

for(i in cb[1]:cb[2]){ # LOOP THROUGH STUDIES (CFB)

for (k in 1:na[i]) { # LOOP THROUGH ARMS

# SE and variances for CFB

b.se[i,k] <- base_SD[i,k]/sqrt(arm_n[i,k])

b.var[i,k] <- pow(b.se[i,k],2)

p.se[i,k] <- post_SD[i,k]/sqrt(arm_n[i,k])

p.var[i,k] <- pow(p.se[i,k],2)

c.se[i,k] <- cfb_SD[i,k]/sqrt(arm_n[i,k])

c.var[i,k] <- pow(c.se[i,k],2)

# Outcome measure: change from baseline (requires baseline)

yC[i,k,1] <- base_m[i,k]

yC[i,k,2] <- cfb[i,k]

phiC[i,k,1] <- ( phiB[i,k] ) * SD_pooled[i] ## study SD

phiC[i,k,2] <- ( theta[i,k] ) * SD_pooled[i] ## study SD

# Likelihood: bivariate Normal

yC[i,k,1:2] ~ dmnorm(phiC[i,k,1:2], sigmaInv[i, k, 1:2, 1:2])

# Precision matrix for mvnorm

sigma[i, k, 1, 1] <- b.var[i,k]

sigma[i, k, 1, 2] <- ( corr * b.se[i,k] * p.se[i,k] )

sigma[i, k, 2, 1] <- ( corr * b.se[i,k] * p.se[i,k] )

sigma[i, k, 2, 2] <- c.var[i,k]

sigmaInv[i, k, 1:2, 1:2] <- inverse(sigma[i, k, 1:2, 1:2])

# Deviance: Mahalanobis distance for trial i (baseline and CFB data)

for (j in 1:2) { ## n of dimensions of mvnorm (i.e. bivariate)

res[i, k, j] <- yC[i, k, j] - phiC[i, k, j]

temp[i, k, j] <- inprod(sigmaInv[i, k, j, 1:2], res[i, k, 1:2])

}

Msq[i,k] <- inprod(res[i, k, 1:2], temp[i, k, 1:2])

M[i,k] <- sqrt(Msq[i,k])

dev[i,k] <- Msq[i,k]

} ## END ARM LOOP

} # END STUDY LOOP FOR CFB DATA

### SECTION 6 - estimation specific to studies reporting baseline and follow-up scores

for(i in pp[1]:pp[2] ) { ## LOOP THROUGH STUDIES (baseline and follow-up)

for (k in 1:na[i]) { ## LOOP THROUGH ARMS

# SE and variances at baseline and follow-up

b.se[i,k] <- base_SD[i,k]/sqrt(arm_n[i,k])

b.var[i,k] <- pow(b.se[i,k],2)

p.se[i,k] <- post_SD[i,k]/sqrt(arm_n[i,k])

p.var[i,k] <- pow(p.se[i,k],2)

# Outcome measure: baseline and follow-up means standardised by study SD

ypp[i,k,1] <- base_m[i,k]

ypp[i,k,2] <- post_m[i,k]

phiPP[i,k,1] <- ( phiB[i,k] ) * SD_pooled[i] ## study SD

phiPP[i,k,2] <- ( phiB[i,k] + theta[i,k] ) * SD_pooled[i] ## study SD

# Likelihood: bivariate Normal

ypp[i,k,1:2] ~ dmnorm(phiPP[i,k,1:2], sigmaInv[i, k, 1:2, 1:2])

# Precision matrix for mvnorm

sigma[i, k, 1, 1] <- b.var[i,k]

sigma[i, k, 1, 2] <- ( corr * b.se[i,k] * p.se[i,k] )

sigma[i, k, 2, 1] <- ( corr * b.se[i,k] * p.se[i,k] )

sigma[i, k, 2, 2] <- p.var[i,k]

sigmaInv[i, k, 1:2, 1:2] <- inverse(sigma[i, k, 1:2, 1:2])

# Deviance: Mahalanobis distance for trial i (baseline and follow-up data)

for (j in 1:2) { ## n of dimensions of mvnorm (i.e. bivariate)

res[i, k, j] <- ypp[i, k, j] - phiPP[i, k, j]

temp[i, k, j] <- inprod(sigmaInv[i, k, j, 1:2], res[i, k, 1:2])

}

Msq[i,k] <- inprod(res[i, k, 1:2], temp[i, k, 1:2])

M[i,k] <- sqrt(Msq[i,k])

dev[i,k] <- Msq[i,k]

} ## END ARM LOOP

} ## END STUDY LOOP (baseline and follow-up data)

### SECTION 7 - estimation specific to studies reporting follow-up scores

for(i in pt[1]:pt[2]){ ## LOOP THROUGH STUDIES (follow-up data)

for (k in 1:na[i]) { ## LOOP THROUGH ARMS

# SE

p.se[i,k] <- post_SD[i,k]/sqrt(arm_n[i,k]) ## SE

p.prec[i,k] <- pow(p.se[i,k],-2) ## precision

# Outcome measure: post-treatment mean

yp[i,k] <- post_m[i,k]

phip[i,k] <- theta[i,k] * SD_pooled[i] ## theta standardised by study SD

# Likelihood: univariate Normal

yp[i,k] ~ dnorm(phip[i,k], p.prec[i,k])

# Deviance: contribution for post-treatment mean

dev[i,k] <- (yp[i,k]- phip[i, k])*(yp[i,k]- phip[i, k])*p.prec[i,k]

} # END ARM LOOP

} ## END STUDY LOOP (follow-up data)

## SECTION 8 - Calculate mean pooled SD for each study, length=ns

for(g in 1:ns) { # LOOP THROUGH STUDIES

for(h in 1: na[g]) { # LOOP THROUGH ARMS

pm.step1[g,h] <- ( (arm_n[g,h] - 1) * pow(base_SD[g,h], 2) )

pm.step2[g,h] <- ( base_m[g,h] * arm_n[g,h] )

}

SD_pooled[g] <- sqrt( sum(pm.step1[g, 1:na[g]]) / (n[g] - na[g]) )

pooledM[g] <- sum(pm.step2[g, 1:na[g]] ) / n[g]

n[g] <- sum(arm_n[g,1: na[g]])

} ## END LOOP (pooled means)

## SECTION 9 - Scale-specific means, length=nScales

## Estimates the mean pooled baseline SD for each scale

for(j in 1:nScales){ ## LOOP THROUGH SCALES

for(h in 1:ns) { ## LOOP THROUGH STUDIES (h)

pooledSD_array[h,j] <- SD_pooled[h] * equals(scale[h], j) ## select correct rows

scaleCount[h,j] <- equals(scale[h], j) ## count instances of scale

} ## END STUDY LOOP (h)

# Mean pooled SD for scale j

pooledSD_scale[j] <- sum(pooledSD_array[,j]) / sum(scaleCount[,j])

} ## END LOOP (scale means)

### SECTION 10 - dummy variables so that same dataset may be used for all models

dv[1] <- atrt[1]

dv[2] <- studyID[1]

dv[3] <- scale[1]

dv[4] <- type[1]

} # *** PROGRAM ENDS

## Study-SMD, Meta-Regression

Noting that back-transformations of treatment effects (d) and between-study SD (sd) require the mean pooled SD for the HAMD-17 scale.

Model{ # *** PROGRAM STARTS

### SECTION 1 - specifications and transformations, length=1

d[1] <- 0 ## treatment effect is zero for reference treatment

beta[1] <- 0 ## effect of baseline severity is zero for placebo arms

tau <- pow(sd,-2) ## between-trial precision = (1/between-trial variance)

centSev <- sum(w_sev[])/sum(n[]) ## mean of baseline severity, for centring

T[1] <- A[2] ## A[2] is mean follow-up score on HAMD-17 scale, reference placebo arm

baselineSD <- pooledSD_scale[2] ## Pooled SD for baseline (HAMD-17)

sdNat <- sd * baselineSD ## Between-study SD on the natural scale rather than SD scale

totresdev <- sum(resdev[]) ## Total Residual Deviance

dPred[1] <- 0 ## treatment effect is zero for reference treatment

### SECTION 2 - priors

sd ~ dunif(0,5) # vague prior for between-trial SD

B ~ dnorm(0, 0.001) # vague prior for meta-regression coefficient

for (k in 2:nt){ d[k] ~ dnorm(0,.0001) } ## vague priors for treatment effects

for (i in 1:ns){ mu[i] ~ dnorm(0, 0.001) ## vague priors for study-level means

for (k in 1:na[i]){ phiB[i,k] ~ dnorm(0, 0.0001) ## vague priors for baselines

}

}

### SECTION 3 - treatment effects and transformations

# Calculate absolute differences from relative effects, length=nt-1

for (k in 2:nt){

beta[k] <- B * atrt[k] ## common covariate effect (B) multiplied by whether t was active

T[k] <- A[2] + dNatAll[1,k] ## Mean at follow-up on chosen scale at mean severity

MD[k] <- T[k] - T[1] ## treatment effect as mean difference on chosen scale

RoM[k] <- T[k] / T[1] ## treatment effect as ratio on chosen scale

dPred[k] ~ dnorm(d[k], tau)

}

# Calculate all relative treatment differences, array [nt-1, nt]

for (c in 1:(nt-1)) {

for (k in (c+1):nt) {

dAll[c,k] <- d[k] - d[c]

dNatAll[c,k] <- baselineSD*(dAll[c,k])

dPredAll[c,k] <- dPred[k] - dPred[c]

dPredNatAll[c,k] <- baselineSD*(dPredAll[c,k])

}

}

# Ranking on relative scale, length=nt

for (k in 1:nt) {

rk[k] <- rank(d[],k) ## assumes events are "bad" / negative d values are good

best[k] <- equals(rk[k],1) ## probability that treat k is best

for (h in 1:nt){ prob[h,k] <- equals(rk[k],h) } ## probability that treat k is h-th best

}

### SECTION 4 - network meta-analysis model, length = ns (with arm loops within)

for(i in 1:ns){ # LOOP THROUGH STUDIES

w[i,1] <- 0 ## adjustment for multi-arm trials is zero for control arm

delta[i,1] <- 0 ## treatment effect is zero for control arm

resdev[i] <- sum(dev[i,1:na[i]]) ## summed residual deviance for study i

## Estimate standardised baseline severity for meta-regression models, centred by weighted mean

b_sev[i] <- pooledM[i] / pooledSD_scale[scale[i]] ## standardising baseline severity

b_sev_c[i] <- b_sev[i] - centSev ## centring baseline severity by mean centSev

w_sev[i] <- b_sev[i]*n[i] ## weighting baseline severity by study size

for(k in 1:na[i]) { ## LOOP THROUGH ARMS

## model for linear predictor (including meta-reg term for severity)

theta[i,k] <- mu[i] + delta[i,k] + (beta[t[i,k]]-beta[t[i,1]]) * b_sev_c[i]

} ## CLOSE ARM LOOP

for (k in 2:na[i]) { ## LOOP THROUGH ARMS >=2

## treatment difference in study i, arm k

delta[i,k] ~ dnorm(md[i,k],taud[i,k])

## mean and precision of distributions (with multi-arm trial correction)

md[i,k] <- d[t[i,k]] - d[t[i,1]] + sw[i,k]

taud[i,k] <- tau *2*(k-1)/k

## adjustment and cumulative adjustment for multi-arm RCTs

w[i,k] <- (delta[i,k] - d[t[i,k]] + d[t[i,1]])

sw[i,k] <- sum(w[i,1:k-1])/(k-1)

} # END ARM LOOP >=2

} # END STUDY LOOP

### SECTION 5 - estimation specific to studies reporting CFB

for(i in cb[1]:cb[2]){ # LOOP THROUGH STUDIES (CFB)

for (k in 1:na[i]) { # LOOP THROUGH ARMS

# SE and variances for CFB

b.se[i,k] <- base_SD[i,k]/sqrt(arm_n[i,k])

b.var[i,k] <- pow(b.se[i,k],2)

p.se[i,k] <- post_SD[i,k]/sqrt(arm_n[i,k])

p.var[i,k] <- pow(p.se[i,k],2)

c.se[i,k] <- cfb_SD[i,k]/sqrt(arm_n[i,k])

c.var[i,k] <- pow(c.se[i,k],2)

# Outcome measure: change from baseline (requires baseline)

yC[i,k,1] <- base_m[i,k]

yC[i,k,2] <- cfb[i,k]

phiC[i,k,1] <- ( phiB[i,k] ) * SD_pooled[i] ## study SD

phiC[i,k,2] <- ( theta[i,k] ) * SD_pooled[i] ## study SD

# Likelihood: bivariate Normal

yC[i,k,1:2] ~ dmnorm(phiC[i,k,1:2], sigmaInv[i, k, 1:2, 1:2])

# Precision matrix for mvnorm

sigma[i, k, 1, 1] <- b.var[i,k]

sigma[i, k, 1, 2] <- ( corr * b.se[i,k] * p.se[i,k] )

sigma[i, k, 2, 1] <- ( corr * b.se[i,k] * p.se[i,k] )

sigma[i, k, 2, 2] <- c.var[i,k]

sigmaInv[i, k, 1:2, 1:2] <- inverse(sigma[i, k, 1:2, 1:2])

# Deviance: Mahalanobis distance for trial i (baseline and CFB data)

for (j in 1:2) { ## n of dimensions of mvnorm (i.e. bivariate)

res[i, k, j] <- yC[i, k, j] - phiC[i, k, j]

temp[i, k, j] <- inprod(sigmaInv[i, k, j, 1:2], res[i, k, 1:2])

}

Msq[i,k] <- inprod(res[i, k, 1:2], temp[i, k, 1:2])

M[i,k] <- sqrt(Msq[i,k])

dev[i,k] <- Msq[i,k]

} ## END ARM LOOP

} # END STUDY LOOP FOR CFB DATA

### SECTION 6 - estimation specific to studies reporting baseline and follow-up scores

for(i in pp[1]:pp[2] ) { ## LOOP THROUGH STUDIES (baseline and follow-up)

for (k in 1:na[i]) { ## LOOP THROUGH ARMS

# SE and variances at baseline and follow-up

b.se[i,k] <- base_SD[i,k]/sqrt(arm_n[i,k])

b.var[i,k] <- pow(b.se[i,k],2)

p.se[i,k] <- post_SD[i,k]/sqrt(arm_n[i,k])

p.var[i,k] <- pow(p.se[i,k],2)

# Outcome measure: baseline and follow-up means standardised by study SD

ypp[i,k,1] <- base_m[i,k]

ypp[i,k,2] <- post_m[i,k]

phiPP[i,k,1] <- ( phiB[i,k] ) * SD_pooled[i] ## study SD

phiPP[i,k,2] <- ( phiB[i,k] + theta[i,k] ) * SD_pooled[i] ## study SD

# Likelihood: bivariate Normal

ypp[i,k,1:2] ~ dmnorm(phiPP[i,k,1:2], sigmaInv[i, k, 1:2, 1:2])

# Precision matrix for mvnorm

sigma[i, k, 1, 1] <- b.var[i,k]

sigma[i, k, 1, 2] <- ( corr * b.se[i,k] * p.se[i,k] )

sigma[i, k, 2, 1] <- ( corr * b.se[i,k] * p.se[i,k] )

sigma[i, k, 2, 2] <- p.var[i,k]

sigmaInv[i, k, 1:2, 1:2] <- inverse(sigma[i, k, 1:2, 1:2])

# Deviance: Mahalanobis distance for trial i (baseline and follow-up data)

for (j in 1:2) { ## n of dimensions of mvnorm (i.e. bivariate)

res[i, k, j] <- ypp[i, k, j] - phiPP[i, k, j]

temp[i, k, j] <- inprod(sigmaInv[i, k, j, 1:2], res[i, k, 1:2])

}

Msq[i,k] <- inprod(res[i, k, 1:2], temp[i, k, 1:2])

M[i,k] <- sqrt(Msq[i,k])

dev[i,k] <- Msq[i,k]

} ## END ARM LOOP

} ## END STUDY LOOP (baseline and follow-up data)

### SECTION 7 - estimation specific to studies reporting follow-up scores

for(i in pt[1]:pt[2]){ ## LOOP THROUGH STUDIES (follow-up data)

for (k in 1:na[i]) { ## LOOP THROUGH ARMS

# SE

p.se[i,k] <- post_SD[i,k]/sqrt(arm_n[i,k]) ## SE

p.prec[i,k] <- pow(p.se[i,k],-2) ## precision

# Outcome measure: post-treatment mean

yp[i,k] <- post_m[i,k]

phip[i,k] <- theta[i,k] * SD_pooled[i] ## theta standardised by study SD

# Likelihood: univariate Normal

yp[i,k] ~ dnorm(phip[i,k], p.prec[i,k])

# Deviance: contribution for post-treatment mean

dev[i,k] <- (yp[i,k]-phip[i, k])*(yp[i,k]-phip[i, k])*p.prec[i,k]

} # END ARM LOOP

} ## END STUDY LOOP (follow-up data)

## SECTION 8 - Calculate pooled mean for each study, length=ns

for(g in 1:ns) { # LOOP THROUGH STUDIES

for(h in 1: na[g]) { # LOOP THROUGH ARMS

pm.step1[g,h] <- ( (arm_n[g,h] - 1) * pow(base_SD[g,h], 2) )

pm.step2[g,h] <- ( base_m[g,h] * arm_n[g,h] )

}

SD_pooled[g] <- sqrt( sum(pm.step1[g, 1:na[g]]) / (n[g] - na[g]) )

pooledM[g] <- sum(pm.step2[g, 1:na[g]] ) / n[g]

n[g] <- sum(arm_n[g,1: na[g]])

} ## END LOOP (pooled means)

## SECTION 9 - Scale-specific means, length=nScales

for(j in 1:nScales){ ## LOOP THROUGH SCALES

for(h in 1:ns) { ## LOOP THROUGH STUDIES (h)

pooledSD_array[h,j] <- SD_pooled[h] * equals(scale[h], j) ## select correct rows

scaleCount[h,j] <- equals(scale[h], j) ## count instances of scale

} ## END STUDY LOOP (h)

# Mean pooled SD for scale j

pooledSD_scale[j] <- sum(pooledSD_array[,j]) / sum(scaleCount[,j])

} ## END LOOP (scale means)

### SECTION 10 - dummy variables so that same dataset may be used for all models

dv[1] <- atrt[1]

dv[2] <- studyID[1]

dv[3] <- scale[1]

dv[4] <- type[1]

} # *** PROGRAM ENDS

## Scale-SMD

## Scale-SMD

model{ # *** PROGRAM STARTS

### SECTION 1 - specifications and transformations, length=1

d[1] <- 0 ## treatment effect is zero for reference treatment

dPred[1] <- 0 ## treatment effect is zero for reference treatment

tau <- pow(sd,-2) ## between-trial precision = (1/between-trial variance)

T[1] <- A[2] ## A[2] is mean follow-up score on HAMD-17 scale, reference placebo arm

baselineSD <- pooledSD_scale[2] ## Pooled SD for baseline (HAMD-17)

sdNat <- sd * baselineSD ## Between-study SD on the natural scale rather than SD scale

totresdev <- sum(resdev[]) ## Total Residual Deviance

### SECTION 2 - priors

sd ~ dunif(0,5) # vague prior for between-trial SD

for (k in 2:nt){ d[k] ~ dnorm(0,.0001) } ## vague priors for treatment effects

for (i in 1:ns){ mu[i] ~ dnorm(0, 0.001) ## vague priors for study-level means

for (k in 1:na[i]){ phiB[i,k] ~ dnorm(0, 0.0001) ## vague priors for baselines

}

}

### SECTION 3 - treatment effects and transformations

# Calculate absolute differences from relative effects, length=nt-1

for (k in 2:nt){

T[k] <- A[2] + dNatAll[1,k] ## Mean at follow-up on chosen scale at mean severity

MD[k] <- T[k] - T[1] ## treatment effect as mean difference on chosen scale

RoM[k] <- T[k] / T[1] ## treatment effect as ratio on chosen scale

dPred[k] ~ dnorm(d[k], tau)

}

# Calculate all relative treatment differences, array [nt-1, nt]

for (c in 1:(nt-1)) {

for (k in (c+1):nt) {

dAll[c,k] <- d[k] - d[c]

dNatAll[c,k] <- baselineSD*(dAll[c,k])

dPredAll[c,k] <- dPred[k] - dPred[c]

dPredNatAll[c,k] <- baselineSD*(dPredAll[c,k])

}

}

# Ranking on relative scale, length=nt

for (k in 1:nt) {

rk[k] <- rank(d[],k) ## assumes events are "bad" / negative d values are good

best[k] <- equals(rk[k],1) ## probability that treat k is best

for (h in 1:nt){ prob[h,k] <- equals(rk[k],h) } ## probability that treat k is h-th best

}

### SECTION 4 - network meta-analysis model, length = ns (with arm loops within)

for(i in 1:ns){ # LOOP THROUGH STUDIES

w[i,1] <- 0 ## adjustment for multi-arm trials is zero for control arm

delta[i,1] <- 0 ## treatment effect is zero for control arm

resdev[i] <- sum(dev[i,1:na[i]]) ## summed residual deviance for study i

for(k in 1:na[i]) { ## LOOP THROUGH ARMS

## model for linear predictor

theta[i,k] <- mu[i] + delta[i,k]

} ## CLOSE ARM LOOP

for (k in 2:na[i]) { ## LOOP THROUGH ARMS >=2

## treatment difference in study i, arm k

delta[i,k] ~ dnorm(md[i,k],taud[i,k])

## mean and precision of distributions (with multi-arm trial correction)

md[i,k] <- d[t[i,k]] - d[t[i,1]] + sw[i,k]

taud[i,k] <- tau *2*(k-1)/k

## adjustment and cumulative adjustment for multi-arm RCTs

w[i,k] <- (delta[i,k] - d[t[i,k]] + d[t[i,1]])

sw[i,k] <- sum(w[i,1:k-1])/(k-1)

} # END ARM LOOP >=2

} # END STUDY LOOP

### SECTION 5 - estimation specific to studies reporting CFB

for(i in cb[1]:cb[2]){ # LOOP THROUGH STUDIES (CFB)

for (k in 1:na[i]) { # LOOP THROUGH ARMS

# SE and variances for CFB

b.se[i,k] <- base_SD[i,k]/sqrt(arm_n[i,k])

b.var[i,k] <- pow(b.se[i,k],2)

p.se[i,k] <- post_SD[i,k]/sqrt(arm_n[i,k])

p.var[i,k] <- pow(p.se[i,k],2)

c.se[i,k] <- cfb_SD[i,k]/sqrt(arm_n[i,k])

c.var[i,k] <- pow(c.se[i,k],2)

# Outcome measure: change from baseline (requires baseline)

yC[i,k,1] <- base_m[i,k]

yC[i,k,2] <- cfb[i,k]

phiC[i,k,1] <- ( phiB[i,k] ) *pooledSD_scale[scale[i]] ## scale SD

phiC[i,k,2] <- ( theta[i,k] ) * pooledSD_scale[scale[i]] ## scale SD

# Likelihood: bivariate Normal

yC[i,k,1:2] ~ dmnorm(phiC[i,k,1:2], sigmaInv[i, k, 1:2, 1:2])

# Precision matrix for mvnorm

sigma[i, k, 1, 1] <- b.var[i,k]

sigma[i, k, 1, 2] <- ( corr * b.se[i,k] * p.se[i,k] )

sigma[i, k, 2, 1] <- ( corr * b.se[i,k] * p.se[i,k] )

sigma[i, k, 2, 2] <- c.var[i,k]

sigmaInv[i, k, 1:2, 1:2] <- inverse(sigma[i, k, 1:2, 1:2])

# Deviance: Mahalanobis distance for trial i (baseline and CFB data)

for (j in 1:2) { ## n of dimensions of mvnorm (i.e. bivariate)

res[i, k, j] <- yC[i, k, j] - phiC[i, k, j]

temp[i, k, j] <- inprod(sigmaInv[i, k, j, 1:2], res[i, k, 1:2])

}

Msq[i,k] <- inprod(res[i, k, 1:2], temp[i, k, 1:2])

M[i,k] <- sqrt(Msq[i,k])

dev[i,k] <- Msq[i,k]

} ## END ARM LOOP

} # END STUDY LOOP FOR CFB DATA

### SECTION 6 - estimation specific to studies reporting baseline and follow-up scores

for(i in pp[1]:pp[2] ) { ## LOOP THROUGH STUDIES (baseline and follow-up)

for (k in 1:na[i]) { ## LOOP THROUGH ARMS

# SE and variances at baseline and follow-up

b.se[i,k] <- base_SD[i,k]/sqrt(arm_n[i,k])

b.var[i,k] <- pow(b.se[i,k],2)

p.se[i,k] <- post_SD[i,k]/sqrt(arm_n[i,k])

p.var[i,k] <- pow(p.se[i,k],2)

# Outcome measure: baseline and post-treatment means standardised by scale SD

ypp[i,k,1] <- base_m[i,k]

ypp[i,k,2] <- post_m[i,k]

phiPP[i,k,1] <- ( phiB[i,k] ) * pooledSD_scale[scale[i]] ## scale SD

phiPP[i,k,2] <- ( phiB[i,k] + theta[i,k] ) * pooledSD_scale[scale[i]] ## scale SD

# Likelihood: bivariate Normal

ypp[i,k,1:2] ~ dmnorm(phiPP[i,k,1:2], sigmaInv[i, k, 1:2, 1:2])

# Precision matrix for mvnorm

sigma[i, k, 1, 1] <- b.var[i,k]

sigma[i, k, 1, 2] <- ( corr * b.se[i,k] * p.se[i,k] )

sigma[i, k, 2, 1] <- ( corr * b.se[i,k] * p.se[i,k] )

sigma[i, k, 2, 2] <- p.var[i,k]

sigmaInv[i, k, 1:2, 1:2] <- inverse(sigma[i, k, 1:2, 1:2])

# Deviance: Mahalanobis distance for trial i (baseline and follow-up data)

for (j in 1:2) { ## n of dimensions of mvnorm (i.e. bivariate)

res[i, k, j] <- ypp[i, k, j] - phiPP[i, k, j]

temp[i, k, j] <- inprod(sigmaInv[i, k, j, 1:2], res[i, k, 1:2])

}

Msq[i,k] <- inprod(res[i, k, 1:2], temp[i, k, 1:2])

M[i,k] <- sqrt(Msq[i,k])

dev[i,k] <- Msq[i,k]

} ## END ARM LOOP

} ## END STUDY LOOP (baseline and follow-up data)

### SECTION 7 - estimation specific to studies reporting follow-up scores

for(i in pt[1]:pt[2]){ ## LOOP THROUGH STUDIES (follow-up data)

for (k in 1:na[i]) { ## LOOP THROUGH ARMS

# SE

p.se[i,k] <- post_SD[i,k]/sqrt(arm_n[i,k]) ## SE

p.prec[i,k] <- pow(p.se[i,k],-2) ## precision

# Outcome measure: post-treatment mean

yp[i,k] <- post_m[i,k]

phip[i,k] <- theta[i,k] * pooledSD_scale[scale[i]]

# Likelihood: univariate Normal

yp[i,k] ~ dnorm(phip[i,k], p.prec[i,k])

# Deviance: contribution for post-treatment mean

dev[i,k] <- (yp[i,k]-phip[i, k])*(yp[i,k]-phip[i, k])*p.prec[i,k]

} # END ARM LOOP

} ## END STUDY LOOP (follow-up data)

## SECTION 8 - Calculate pooled mean for each study, length=ns

for(g in 1:ns) { # LOOP THROUGH STUDIES

for(h in 1: na[g]) { # LOOP THROUGH ARMS

pm.step1[g,h] <- ( (arm_n[g,h] - 1) * pow(base_SD[g,h], 2) )

pm.step2[g,h] <- ( base_m[g,h] * arm_n[g,h] )

}

SD_pooled[g] <- sqrt( sum(pm.step1[g, 1:na[g]]) / (n[g] - na[g]) )

pooledM[g] <- sum(pm.step2[g, 1:na[g]] ) / n[g]

n[g] <- sum(arm_n[g,1: na[g]])

} ## END LOOP (pooled means)

## SECTION 9 - Scale-specific means, length=nScales

for(j in 1:nScales){ ## LOOP THROUGH SCALES

for(h in 1:ns) { ## LOOP THROUGH STUDIES (h)

pooledSD_array[h,j] <- SD_pooled[h] * equals(scale[h], j) ## select correct rows

scaleCount[h,j] <- equals(scale[h], j) ## count instances of scale

} ## END STUDY LOOP (h)

# Mean pooled SD for scale j

pooledSD_scale[j] <- sum(pooledSD_array[,j]) / sum(scaleCount[,j])

} ## END LOOP (scale means)

### SECTION 10 - dummy variables so that same dataset may be used for all models

dv[1] <- atrt[1]

dv[2] <- studyID[1]

dv[3] <- scale[1]

dv[4] <- type[1]

} # *** PROGRAM ENDS

## Scale-SMD, Meta-Regression

## Scale-SMD, MR

model{ # *** PROGRAM STARTS

### SECTION 1 - specifications and transformations, length=1

d[1] <- 0 ## treatment effect is zero for reference treatment

dPred[1] <- 0 ## treatment effect is zero for reference treatment

beta[1] <- 0 ## effect of baseline severity is zero for placebo arms

tau <- pow(sd,-2) ## between-trial precision = (1/between-trial variance)

centSev <- sum(w_sev[])/sum(n[]) ## mean of baseline severity, for centring

T[1] <- A[2] ## A[2] is mean follow-up score on HAMD-17 scale, reference placebo arm

baselineSD <- pooledSD_scale[2] ## Pooled SD for baseline (HAMD-17)

sdNat <- sd * baselineSD ## Between-study SD on the natural scale rather than SD scale

totresdev <- sum(resdev[]) ## Total Residual Deviance

### SECTION 2 - priors

sd ~ dunif(0,5) # vague prior for between-trial SD

B ~ dnorm(0, 0.001) # vague prior for meta-regression coefficient

for (k in 2:nt){ d[k] ~ dnorm(0,.0001) } ## vague priors for treatment effects

for (i in 1:ns){ mu[i] ~ dnorm(0, 0.001) ## vague priors for study-level means

for (k in 1:na[i]){ phiB[i,k] ~ dnorm(0, 0.0001) ## vague priors for baselines

}

}

### SECTION 3 - treatment effects and transformations

# Calculate absolute differences from relative effects, length=nt-1

for (k in 2:nt){

beta[k] <- B * atrt[k] ## common covariate effect (B) multiplied by whether t was active

T[k] <- A[2] + dNatAll[1,k] ## Mean at follow-up on chosen scale at mean severity

MD[k] <- T[k] - T[1] ## treatment effect as mean difference on chosen scale

RoM[k] <- T[k] / T[1] ## treatment effect as ratio on chosen scale

dPred[k] ~ dnorm(d[k], tau)

}

# Calculate all relative treatment differences, array [nt-1, nt]

for (c in 1:(nt-1)) {

for (k in (c+1):nt) {

dAll[c,k] <- d[k] - d[c]

dNatAll[c,k] <- baselineSD*(dAll[c,k])

dPredAll[c,k] <- dPred[k] - dPred[c]

dPredNatAll[c,k] <- baselineSD*(dPredAll[c,k])

}

}

# Ranking on relative scale, length=nt

for (k in 1:nt) {

rk[k] <- rank(d[],k) ## assumes events are "bad" / negative d values are good

best[k] <- equals(rk[k],1) ## probability that treat k is best

for (h in 1:nt){ prob[h,k] <- equals(rk[k],h) } ## probability that treat k is h-th best

}

### SECTION 4 - network meta-analysis model, length = ns (with arm loops within)

for(i in 1:ns){ # LOOP THROUGH STUDIES

w[i,1] <- 0 ## adjustment for multi-arm trials is zero for control arm

delta[i,1] <- 0 ## treatment effect is zero for control arm

resdev[i] <- sum(dev[i,1:na[i]]) ## summed residual deviance for study i

## Estimate standardised baseline severity for meta-regression models, centred by weighted mean

b_sev[i] <- pooledM[i] / pooledSD_scale[scale[i]] ## standardising baseline severity

b_sev_c[i] <- b_sev[i] - centSev ## centring baseline severity by mean centSev

w_sev[i] <- b_sev[i]*n[i] ## weighting baseline severity by study size

for(k in 1:na[i]) { ## LOOP THROUGH ARMS

## model for linear predictor (including meta-reg term for severity)

theta[i,k] <- mu[i] + delta[i,k] + (beta[t[i,k]]-beta[t[i,1]]) * b_sev_c[i]

} ## CLOSE ARM LOOP

for (k in 2:na[i]) { ## LOOP THROUGH ARMS >=2

## treatment difference in study i, arm k

delta[i,k] ~ dnorm(md[i,k],taud[i,k])

## mean and precision of distributions (with multi-arm trial correction)

md[i,k] <- d[t[i,k]] - d[t[i,1]] + sw[i,k]

taud[i,k] <- tau *2*(k-1)/k

## adjustment and cumulative adjustment for multi-arm RCTs

w[i,k] <- (delta[i,k] - d[t[i,k]] + d[t[i,1]])

sw[i,k] <- sum(w[i,1:k-1])/(k-1)

} # END ARM LOOP >=2

} # END STUDY LOOP

### SECTION 5 - estimation specific to studies reporting CFB

for(i in cb[1]:cb[2]){ # LOOP THROUGH STUDIES (CFB)

for (k in 1:na[i]) { # LOOP THROUGH ARMS

# SE and variances for CFB

b.se[i,k] <- base_SD[i,k]/sqrt(arm_n[i,k])

b.var[i,k] <- pow(b.se[i,k],2)

p.se[i,k] <- post_SD[i,k]/sqrt(arm_n[i,k])

p.var[i,k] <- pow(p.se[i,k],2)

c.se[i,k] <- cfb_SD[i,k]/sqrt(arm_n[i,k])

c.var[i,k] <- pow(c.se[i,k],2)

# Outcome measure: change from baseline (requires baseline)

yC[i,k,1] <- base_m[i,k]

yC[i,k,2] <- cfb[i,k]

phiC[i,k,1] <- ( phiB[i,k] ) * pooledSD_scale[scale[i]] ## scale SD

phiC[i,k,2] <- ( theta[i,k] ) * pooledSD_scale[scale[i]] ## scale SD

# Likelihood: bivariate Normal

yC[i,k,1:2] ~ dmnorm(phiC[i,k,1:2], sigmaInv[i, k, 1:2, 1:2])

# Precision matrix for mvnorm

sigma[i, k, 1, 1] <- b.var[i,k]

sigma[i, k, 1, 2] <- ( corr * b.se[i,k] * p.se[i,k] )

sigma[i, k, 2, 1] <- ( corr * b.se[i,k] * p.se[i,k] )

sigma[i, k, 2, 2] <- c.var[i,k]

sigmaInv[i, k, 1:2, 1:2] <- inverse(sigma[i, k, 1:2, 1:2])

# Deviance: Mahalanobis distance for trial i (baseline and CFB data)

for (j in 1:2) { ## n of dimensions of mvnorm (i.e. bivariate)

res[i, k, j] <- yC[i, k, j] - phiC[i, k, j]

temp[i, k, j] <- inprod(sigmaInv[i, k, j, 1:2], res[i, k, 1:2])

}

Msq[i,k] <- inprod(res[i, k, 1:2], temp[i, k, 1:2])

M[i,k] <- sqrt(Msq[i,k])

dev[i,k] <- Msq[i,k]

} ## END ARM LOOP

} # END STUDY LOOP FOR CFB DATA

### SECTION 6 - estimation specific to studies reporting baseline and follow-up scores

for(i in pp[1]:pp[2] ) { ## LOOP THROUGH STUDIES (baseline and follow-up)

for (k in 1:na[i]) { ## LOOP THROUGH ARMS

# SE and variances at baseline and follow-up

b.se[i,k] <- base_SD[i,k]/sqrt(arm_n[i,k])

b.var[i,k] <- pow(b.se[i,k],2)

p.se[i,k] <- post_SD[i,k]/sqrt(arm_n[i,k])

p.var[i,k] <- pow(p.se[i,k],2)

# Outcome measure: baseline and post-treatment means standardised by scale SD

ypp[i,k,1] <- base_m[i,k]

ypp[i,k,2] <- post_m[i,k]

phiPP[i,k,1] <- ( phiB[i,k] ) * pooledSD_scale[scale[i]] ## scale SD

phiPP[i,k,2] <- ( phiB[i,k] + theta[i,k] ) * pooledSD_scale[scale[i]] ## scale SD

# Likelihood: bivariate Normal

ypp[i,k,1:2] ~ dmnorm(phiPP[i,k,1:2], sigmaInv[i, k, 1:2, 1:2])

# Precision matrix for mvnorm

sigma[i, k, 1, 1] <- b.var[i,k]

sigma[i, k, 1, 2] <- ( corr * b.se[i,k] * p.se[i,k] )

sigma[i, k, 2, 1] <- ( corr * b.se[i,k] * p.se[i,k] )

sigma[i, k, 2, 2] <- p.var[i,k]

sigmaInv[i, k, 1:2, 1:2] <- inverse(sigma[i, k, 1:2, 1:2])

# Deviance: Mahalanobis distance for trial i (baseline and follow-up data)

for (j in 1:2) { ## n of dimensions of mvnorm (i.e. bivariate)

res[i, k, j] <- ypp[i, k, j] - phiPP[i, k, j]

temp[i, k, j] <- inprod(sigmaInv[i, k, j, 1:2], res[i, k, 1:2])

}

Msq[i,k] <- inprod(res[i, k, 1:2], temp[i, k, 1:2])

M[i,k] <- sqrt(Msq[i,k])

dev[i,k] <- Msq[i,k]

} ## END ARM LOOP

} ## END STUDY LOOP (baseline and follow-up data)

### SECTION 7 - estimation specific to studies reporting follow-up scores

for(i in pt[1]:pt[2]){ ## LOOP THROUGH STUDIES (follow-up data)

for (k in 1:na[i]) { ## LOOP THROUGH ARMS

# SE

p.se[i,k] <- post_SD[i,k]/sqrt(arm_n[i,k]) ## SE

p.prec[i,k] <- pow(p.se[i,k],-2) ## precision

# Outcome measure: post-treatment mean

yp[i,k] <- post_m[i,k]

phip[i,k] <- theta[i,k] * pooledSD_scale[scale[i]]

# Likelihood: univariate Normal

yp[i,k] ~ dnorm(phip[i,k], p.prec[i,k])

# Deviance: contribution for post-treatment mean

dev[i,k] <- (yp[i,k]-phip[i, k])*(yp[i,k]-phip[i, k])*p.prec[i,k]

} # END ARM LOOP

} ## END STUDY LOOP (follow-up data)

## SECTION 8 - Calculate pooled mean for each study, length=ns

for(g in 1:ns) { # LOOP THROUGH STUDIES

for(h in 1: na[g]) { # LOOP THROUGH ARMS

pm.step1[g,h] <- ( (arm_n[g,h] - 1) * pow(base_SD[g,h], 2) )

pm.step2[g,h] <- ( base_m[g,h] * arm_n[g,h] )

}

SD_pooled[g] <- sqrt( sum(pm.step1[g, 1:na[g]]) / (n[g] - na[g]) )

pooledM[g] <- sum(pm.step2[g, 1:na[g]] ) / n[g]

n[g] <- sum(arm_n[g,1: na[g]])

} ## END LOOP (pooled means)

## SECTION 9 - Scale-specific means, length=nScales

for(j in 1:nScales){ ## LOOP THROUGH SCALES

for(h in 1:ns) { ## LOOP THROUGH STUDIES (h)

pooledSD_array[h,j] <- SD_pooled[h] * equals(scale[h], j) ## select correct rows

scaleCount[h,j] <- equals(scale[h], j) ## count instances of scale

} ## END STUDY LOOP (h)

# Mean pooled SD for scale j

pooledSD_scale[j] <- sum(pooledSD_array[,j]) / sum(scaleCount[,j])

} ## END LOOP (scale means)

### SECTION 10 - dummy variables so that same dataset may be used for all models

dv[1] <- atrt[1]

dv[2] <- studyID[1]

dv[3] <- scale[1]

dv[4] <- type[1]

} # *** PROGRAM ENDS

## RoM

## RoM Model

## Normal likelihood, identity link

## Univ. for CFB (on CFB, not baseline)

## Contains T nodes (back-transformed ds)

## Accepts CFB (loop 1), pre/post (loop 2) and post-treatment data (loop 3)

## Updated 17th November 2022 with code to calc all trt comparisons

## Updated 15th November 2023 (!) with predictive interval code

model{ # *** PROGRAM STARTS

### SECTION 1 - specifications and transformations, length=1

totresdev <- sum(resdev[]) #Total Residual Deviance

d[1] <- 0 ## treatment effect on log scale is zero for reference treatment

dRoM[1] <- 1 ## treatment effect as RoM is zero for reference treatment

dPred[1] <- 0

tau <- pow(sd,-2) ## between-trial precision = (1/between-trial variance)

sdExp <- exp(sd) ## Between-study SD exponentiated

### SECTION 2 - priors

sd ~ dunif(0,5) ## vague prior for between-trial SD

for (k in 2:nt){ d[k] ~ dnorm(0,.0001) } ## vague priors for treatment effects

for (i in 1:ns){ mu[i] ~ dnorm(0, 0.001) ## vague priors for study-level means

for (k in 1:na[i]){

#phiB[i,k] ~ dnorm(0, 0.0001) ## vague priors for baseline

phiB[i,k] ~ dunif(0, 100) ## vague priors for baseline

}

}

### SECTION 3 - treatment effects and transformations

# Calculate treatment effect as ratio of means from log ratio of means, length=nt-1

for (k in 2:nt){ dRoM[k] <- exp(d[k])

dPred[k] ~ dnorm(d[k], tau)

}

# Calculate all relative treatment differences, array [nt-1, nt]

for (c in 1:(nt-1)) { #

for (k in (c+1):nt) { dNatAll[c,k] <- T[k,1] - T[c,1]

dPredNatAll[c,k] <- T[k,3] - T[c,3]

}

}

# Ranking on relative scale, length=nt

for (k in 1:nt) {

rk[k] <- rank(d[],k) ## assumes events are "bad" / negative d values are good

best[k] <- equals(rk[k],1) ## probability that treat k is best

for (h in 1:nt){ prob[h,k] <- equals(rk[k],h) } ## probability that treat k is h-th best

}

for (k in 1:nt) {

## treatment effect as difference between CFB on treatment k and reference treatment (placebo)

T[k,1] <- T[k,2] - (A[2] - A[1])

## Absolute effect treatment k is absolute effect on treatment 1 multiplied by RoM for treatment k

T[k,2] <- A[2] * dRoM[k]

## Absolute treatment effect on the CFB for treatment k, with prediction intervals

T[k,3] <- A[2] * exp(dPred[k])

for (n in 1:3){

MD[k,n] <- T[k,n] - T[1,n]

RoM[k,n] <- T[k,n] / T[1,n]

}

}

### SECTION 4 - network meta-analysis model, length = ns (with arm loops within)

for(i in 1:ns){

w[i,1] <- 0 # adjustment for multi-arm trials is zero for control arm

delta[i,1] <- 0 # treatment effect is zero for control arm

resdev[i] <- sum(dev[i,1:na[i]]) # summed residual deviance for study i

for(k in 1:na[i]) { # OPEN ARM LOOP

theta[i,k] <- mu[i] + delta[i,k] # model for linear predictor

} # CLOSE ARM LOOP

for (k in 2:na[i]) { ## LOOP THROUGH ARMS >=2

## treatment difference in study i, arm k

delta[i,k] ~ dnorm(md[i,k],taud[i,k])

## mean and precision of distributions (with multi-arm trial correction)

md[i,k] <- d[t[i,k]] - d[t[i,1]] + sw[i,k]

taud[i,k] <- tau *2*(k-1)/k

## adjustment and cumulative adjustment for multi-arm RCTs

w[i,k] <- (delta[i,k] - d[t[i,k]] + d[t[i,1]])

sw[i,k] <- sum(w[i,1:k-1])/(k-1)

} # END ARM LOOP >=2

} # END STUDY LOOP

### SECTION 5 - estimation specific to studies reporting CFB

for(i in cb[1]:cb[2]){ # LOOP THROUGH STUDIES (CFB)

for (k in 1:na[i]) { # LOOP THROUGH ARMS

# SE and precision for CFB

b.se[i,k] <- base_SD[i,k]/sqrt(arm_n[i,k]) ## baseline SE

b.var[i,k] <- pow(b.se[i,k],2)

p.se[i,k] <- post_SD[i,k]/sqrt(arm_n[i,k]) ## follow-up SE

p.var[i,k] <- pow(p.se[i,k],2)

c.se[i,k] <- cfb_SD[i,k]/sqrt(arm_n[i,k]) ## CFB SE

c.var[i,k] <- pow(c.se[i,k],2)

# Outcome measure: change from baseline (requires baseline to place theta on log scale)

yC[i,k,1] <- base_m[i,k]

yC[i,k,2] <- cfb[i,k]

log(phiC[i,k,1]) <- phiB[i,k]

phiC[i,k,2] <- phiF[i,k] - phiC[i,k,1] #phiB[i,k] ## CFB

# phiC[i,k,2] <- phiF[i,k] - phiB[i,k] ## CFB

log(phiF[i,k]) <- log(phiC[i,k,1]) + theta[i,k] ## Follow-up

# Likelihood: bivariate Normal

yC[i,k,1:2] ~ dmnorm(phiC[i,k,1:2], sigmaInv[i, k, 1:2, 1:2])

# Precision matrix for mvnorm

sigma[i, k, 1, 1] <- b.var[i,k]

sigma[i, k, 1, 2] <- ( corr * b.se[i,k] * p.se[i,k] )

sigma[i, k, 2, 1] <- ( corr * b.se[i,k] * p.se[i,k] )

sigma[i, k, 2, 2] <- c.var[i,k]

sigmaInv[i, k, 1:2, 1:2] <- inverse(sigma[i, k, 1:2, 1:2])

# Deviance: Mahalanobis distance for trial i (baseline and CFB data)

for (j in 1:2) { ## n of dimensions of mvnorm (i.e. bivariate)

res[i, k, j] <- yC[i, k, j] - phiC[i, k, j]

temp[i, k, j] <- inprod(sigmaInv[i, k, j, 1:2], res[i, k, 1:2])

}

Msq[i,k] <- inprod(res[i, k, 1:2], temp[i, k, 1:2])

M[i,k] <- sqrt(Msq[i,k])

dev[i,k] <- Msq[i,k]

} ## END ARM LOOP

} # END STUDY LOOP FOR CFB DATA

### SECTION 6 - estimation specific to studies reporting baseline and follow-up scores

for(i in pp[1]:pp[2] ) { # LOOP THROUGH STUDIES (PREPOST)

for (k in 1:na[i]) { # LOOP THROUGH ARMS

# SE, variance and precision

b.se[i,k] <- base_SD[i,k]/sqrt(arm_n[i,k]) ## baseline SE

b.var[i,k] <- pow(b.se[i,k],2) # calculate variances (baseline)

p.se[i,k] <- post_SD[i,k]/sqrt(arm_n[i,k]) ## follow-up SE

p.var[i,k] <- pow(p.se[i,k],2) # calculate variances (at follow-up)

# Outcome measure: baseline and follow-up means

ypp[i,k,1] <- base_m[i,k]

ypp[i,k,2] <- post_m[i,k]

log(phiPP[i,k,1]) <- phiB[i,k]

log(phiPP[i,k,2]) <- phiB[i,k] + theta[i,k]

# Likelihood: bivariate Normal

ypp[i,k,1:2] ~ dmnorm(phiPP[i,k,1:2], sigmaInv[i, k, 1:2, 1:2])

# Precision matrix for mvnorm (correlation is given as data)

sigma[i, k, 1, 1] <- b.var[i,k]

sigma[i, k, 1, 2] <- ( corr * b.se[i,k] * p.se[i,k] )

sigma[i, k, 2, 1] <- ( corr * b.se[i,k] * p.se[i,k] )

sigma[i, k, 2, 2] <- p.var[i,k]

sigmaInv[i, k, 1:2, 1:2] <- inverse(sigma[i, k, 1:2, 1:2])

# Deviance: Mahalanobis distance for trial i (PP)

for (j in 1:2) { ## n of dimensions of mvnorm (i.e. bivariate)

res[i, k, j] <- ypp[i, k, j] - phiPP[i, k, j]

temp[i, k, j] <- inprod(sigmaInv[i, k, j, 1:2], res[i, k, 1:2])

}

Msq[i,k] <- inprod(res[i, k, 1:2], temp[i, k, 1:2])

M[i,k] <- sqrt(Msq[i,k])

dev[i,k] <- Msq[i,k]

} # END ARM LOOP

} # END STUDY LOOP FOR PP DATA

### SECTION 7 - estimation specific to studies reporting follow-up scores

for(i in pt[1]:pt[2]){ # LOOP THROUGH STUDIES (follow-up mean)

for (k in 1:na[i]) { # LOOP THROUGH ARMS

# SE, variance and precision

p.se[i,k] <- post_SD[i,k]/sqrt(arm_n[i,k])

p.prec[i,k] <- pow(p.se[i,k],-2)

# Outcome measure: follow-up mean (difference between log(mean) is a ratio)

yp[i,k] <- post_m[i,k]

log(phip[i,k]) <- theta[i,k]

# Likelihood: univariate Normal

yp[i,k] ~ dnorm(phip[i,k], p.prec[i,k])

# Deviance: contribution for follow-up mean

dev[i,k] <- (yp[i,k]-phip[i, k])*(yp[i,k]- phip[i, k])*p.prec[i,k]

} # END ARM LOOP

} # END STUDY LOOP FOR PT DATA

### SECTION 8 - dummy variables so that same dataset may be used for all models

dv[1] <- atrt[1]

dv[2] <- studyID[1]

dv[3] <- scale[1]

dv[4] <- type[1]

} # *** PROGRAM ENDS

# **Appendix 3: Sensitivity analysis on the impact of data format**

In our main analysis, the preference for data format followed that recommended for additive measures: i) CFB should be used where reported; ii) where CFB is not reported, baseline and follow-up scores should be used; and iii) where neither CFB nor baseline is reported, follow-up scores can be used (Daly 2014). Whilst the CFB can be calculated from baseline and follow-up measures by assuming the correlation between scores, here only CFB measures reported by the publication qualified as CFB data.

Since all studies included here reported baseline means with standard deviations, studies where the standard deviation of depression score at follow-up was imputed were specified as reporting follow-up scores only.

To assess the impact of the data format we repeated the analyses on a dataset with the following preference order: i) baseline and follow-up scores used where reported; ii) follow-up scores only; and iii) CFB where follow-up scores are not reported. In practice, this meant that studies previously analysed as CFB were reclassified, and their baseline and follow-up values were analysed.

The results of the sensitivity analysis appear in Tables S3, S4, and S5. Scale-SMD and RoM models continue to show greater % shrinkage (lower heterogeneity) than Study-SMD models. However, when comparing on DIC, all models are equivalent (Table S3).

The sensitivity analysis gives very similar results for treatment recommendations compared to the main analysis, with the slight differences between models observed in the main analysis also seen in the sensitivity analysis (Table S5). In particular, in both analyses it appears that there is better discrimination between active treatments under the RoM model. This is evidenced by the smaller number of treatments that were not worse than the best treatment (decision rule 4), and the larger number of treatments that the best treatment was superior to (decision rule 5).

**Table S3.** Model fit statistics for analyses where data format preference differed: total residual deviance (relative to 616 datapoints), DIC and shrinkage. Posterior median of between-studies SD with the 95% credible interval (CrI) on both the linear predictor scale and, for SMD models, on the HAMD-17 scale.

1. **Main analysis: CFB preferred, then baseline & follow-up, follow-up only**

| Model | Dbar | pD | DIC | %  shrinkage | Regression  Coefficient  mean (95% CrI) | Between-study SD  Scale: linear predictor  Median (95% CrI) | Between-study SD  Scale: HAMD-17  Median (95% CrI) |
| --- | --- | --- | --- | --- | --- | --- | --- |
| Study SMD | 625.3 | 538.2 | 1163.6 | 16.73 | - | 0.34 (0.27, 0.41) | 1.35 (1.10, 1.65) |
| Study SMD (MR) | 625.3 | 537.5 | 1162.8 | 16.94 | -0.168  (-0.333, -0.002) | 0.33 (0.27, 0.40) | 1.33 (1.08, 1.62) |
| Scale SMD | 630.6 | 531.7 | 1162.3 | 18.23 | - | 0.29 (0.23, 0.36) | 1.18 (0.93, 1.45) |
| Scale SMD (MR) | 631.3 | 531.7 | 1163.0 | 18.27 | -0.121  (-0.272, 0.030) | 0.29 (0.23, 0.36) | 1.17 (0.92, 1.44) |
| Ratio of Means | 629.0 | 521.9 | 1150.9 | 20.49 | - | 0.09 (0.07, 0.12) | *Not calculable* |

1. **Sensitivity analysis: baseline & follow-up preferred, then follow-up only, CFB**

| Model | Dbar | pD | DIC | %  shrinkage | Regression  Coefficient  mean (95% CrI) | Between-study SD  Scale: linear predictor  Median (95% CrI) | Between-study SD  Scale: HAMD-17  Median (95% CrI) |
| --- | --- | --- | --- | --- | --- | --- | --- |
| Study SMD | 621.0 | 549.0 | 1170.0 | 14.25 | - | 0.37 (0.31, 0.44) | 1.49 (1.24, 1.78) |
| Study SMD (MR) | 620.7 | 547.7 | 1168.4 | 14.59 | -0.199  (-0.371, -0.026) | 0.36 (0.30, 0.43) | 1.46 (1.21, 1.74) |
| Scale SMD | 627.2 | 540.8 | 1167.9 | 16.14 | - | 0.32 (0.26, 0.38) | 1.27 (1.04, 1.54) |
| Scale SMD (MR) | 628.7 | 539.6 | 1168.4 | 16.45 | -0.145  (-0.302, 0.008) | 0.31 (0.25, 0.38) | 1.24 (1.02, 1.51) |
| Ratio of Means | 626.3 | 541.0 | 1167.3 | 16.09 | - | 0.11 (0.09, 0.13) | *Not calculable* |

**Table S4** Treatment effect estimates as mean differences with 95% CrI relative to placebo on the HAMD-17 scale from models where data format preference was baseline and follow-up, then follow-up only, then CFB. Active treatments are sorted by mean estimated treatment effect (relative to placebo), largest first.

| Study SMD | | Study SMD (MR) | | Scale SMD | | Scale SMD (MR) | | RoM | |
| --- | --- | --- | --- | --- | --- | --- | --- | --- | --- |
| Mirtazapine | -3.68 SD=0.62  (-4.91, -2.46) | Mirtazapine | -3.76 SD=0.61  (-4.99, -2.56) | Amitriptyline | -3.35 SD=0.42  (-4.16, -2.53) | Amitriptyline | -3.43 SD=0.42  (-4.24, -2.62) | Amitriptyline | -3.88 SD=0.43  (-4.70, -3.01) |
| Amitriptyline | -3.60 SD=0.45  (-4.48, -2.70) | Amitriptyline | -3.72 SD=0.45  (-4.59, -2.85) | Mirtazapine | -3.34 SD=0.56  (-4.46, -2.25) | Mirtazapine | -3.40 SD=0.55  (-4.48, -2.32) | Mirtazapine | -3.67 SD=0.58  (-4.75, -2.47) |
| Clomipramine | -3.43 SD=0.99  (-5.35, -1.49) | Clomipramine | -3.52 SD=0.97  (-5.42, -1.59) | Clomipramine | -3.15 SD=0.97  (-5.05, -1.26) | Clomipramine | -3.20 SD=0.96  (-5.12, -1.31) | Lofepramine | -3.37 SD=1.45  (-5.87, -0.19) |
| Venlafaxine | -3.21 SD=0.41  (-4.02, -2.41) | Paroxetine | -3.27 SD=0.44  (-4.14, -2.43) | Venlafaxine | -2.98 SD=0.36  (-3.68, -2.27) | Venlafaxine | -3.01 SD=0.36  (-3.72, -2.31) | Venlafaxine | -3.29 SD=0.39  (-4.05, -2.50) |
| Paroxetine | -3.19 SD=0.44  (-4.06, -2.33) | Venlafaxine | -3.26 SD=0.41  (-4.07, -2.47) | Imipramine | -2.80 SD=0.54  (-3.85, -1.74) | Paroxetine | -2.84 SD=0.38  (-3.60, -2.10) | Clomipramine | -3.22 SD=1.12  (-5.24, -0.84) |
| Lofepramine | -3.04 SD=1.41  (-5.80, -0.26) | Lofepramine | -3.15 SD=1.39  (-5.89, -0.40) | Paroxetine | -2.80 SD=0.39  (-3.54, -2.04) | Imipramine | -2.81 SD=0.53  (-3.87, -1.76) | Paroxetine | -3.10 SD=0.43  (-3.92, -2.21) |
| Escitalopram | -2.98 SD=0.36  (-3.68, -2.25) | Duloxetine | -3.01 SD=0.41  (-3.81, -2.21) | Escitalopram | -2.66 SD=0.31  (-3.29, -2.04) | Duloxetine | -2.78 SD=0.36  (-3.49, -2.06) | Duloxetine | -3.04 SD=0.39  (-3.79, -2.27) |
| Duloxetine | -2.80 SD=0.41  (-3.59, -2.00) | Escitalopram | -2.99 SD=0.36  (-3.69, -2.28) | Duloxetine | -2.63 SD=0.36  (-3.34, -1.93) | Escitalopram | -2.67 SD=0.31  (-3.28, -2.06) | Escitalopram | -2.89 SD=0.34  (-3.55, -2.19) |
| Imipramine | -2.79 SD=0.57  (-3.92, -1.65) | Imipramine | -2.82 SD=0.56  (-3.93, -1.70) | Lofepramine | -2.55 SD=1.51  (-5.50, 0.43) | Lofepramine | -2.60 SD=1.50  (-5.59, 0.33) | Imipramine | -2.86 SD=0.58  (-3.94, -1.66) |
| Sertraline | -2.65 SD=0.41  (-3.46, -1.83) | Sertraline | -2.75 SD=0.41  (-3.54, -1.95) | Sertraline | -2.35 SD=0.35  (-3.04, -1.66) | Sertraline | -2.41 SD=0.35  (-3.09, -1.73) | Sertraline | -2.67 SD=0.39  (-3.40, -1.88) |
| Fluoxetine | -2.45 SD=0.34  (-3.13, -1.78) | Fluoxetine | -2.53 SD=0.34  (-3.20, -1.86) | Citalopram | -2.29 SD=0.50  (-3.26, -1.31) | Citalopram | -2.32 SD=0.49  (-3.29, -1.36) | Citalopram | -2.39 SD=0.60  (-3.52, -1.17) |
| Citalopram | -2.42 SD=0.57  (-3.53, -1.31) | Citalopram | -2.50 SD=0.56  (-3.57, -1.43) | Nortriptyline | -2.22 SD=0.78  (-3.75, -0.68) | Nortriptyline | -2.28 SD=0.76  (-3.77, -0.76) | Fluoxetine | -2.37 SD=0.35  (-3.03, -1.67) |
| Nortriptyline | -2.29 SD=0.83  (-3.93, -0.66) | Nortriptyline | -2.29 SD=0.81  (-3.92, -0.72) | Fluoxetine | -2.22 SD=0.30  (-2.81, -1.64) | Fluoxetine | -2.27 SD=0.30  (-2.86, -1.70) | Nortriptyline | -2.24 SD=0.93  (-3.96, -0.33) |
| Trazodone | -1.83 SD=0.69  (-3.15, -0.46) | Trazodone | -1.92 SD=0.68  (-3.24, -0.58) | Trazodone | -1.77 SD=0.57  (-2.88, -0.64) | Trazodone | -1.86 SD=0.58  (-2.98, -0.71) | Trazodone | -1.57 SD=0.74  (-2.92, -0.04) |
| No treatment | -2.45 SD=1.66  (-5.71, 0.79) | No treatment | -1.63 SD=1.67  (-4.88, 1.69) | No treatment | -1.32 SD=1.76  (-4.81, 2.12) | No treatment | -0.68 SD=1.81  (-4.22, 2.86) | No treatment | -1.30 SD=2.28  (-5.11, 3.78) |

**Table S5.** Treatment recommendations on each model from sensitivity analyses, where data format preference was baseline and follow-up, then follow-up only, then CFB. Treatments are listed in order of efficacy, from five decision rules.

| Decision rule | Study SMD | Study SMD Meta-regression | Scale-SMD | Scale-SMD Meta-regression | RoM |
| --- | --- | --- | --- | --- | --- |
| 1. Single best treatment | Mirtazapine | Mirtazapine | Amitriptyline | Amitriptyline | Amitriptyline |
| 2. Treatments better than placebo^1^ | Mirtazapine Amitriptyline  Clomipramine  Venlafaxine  Paroxetine  Escitalopram  Imipramine  Duloxetine  Sertraline  Citalopram  Fluoxetine  Lofepramine  Nortriptyline  Trazodone | Mirtazapine Amitriptyline  Clomipramine  Paroxetine  Venlafaxine  Duloxetine  Escitalopram  Imipramine  Sertraline  Citalopram  Fluoxetine  Lofepramine  Nortriptyline  Trazodone | Amitriptyline  Mirtazapine  Clomipramine  Venlafaxine  Imipramine  Paroxetine  Escitalopram  Duloxetine  Citalopram  Sertraline  Fluoxetine  Nortriptyline  Trazodone | Amitriptyline  Mirtazapine  Clomipramine  Venlafaxine  Imipramine  Duloxetine  Paroxetine  Escitalopram  Citalopram  Sertraline  Fluoxetine  Nortriptyline  Trazodone | Amitriptyline  Mirtazapine  Venlafaxine  Duloxetine  Paroxetine  Imipramine  Escitalopram  Sertraline  Fluoxetine  Citalopram  Clomipramine  Lofepramine  Nortriptyline  Trazodone |
| 3. Treatments better than placebo by 1 unit on HAMD-17^2^ scale | Mirtazapine  Amitriptyline  Clomipramine  Venlafaxine  Paroxetine  Escitalopram  Imipramine  Duloxetine  Sertraline  Citalopram  Fluoxetine | Mirtazapine  Amitriptyline  Clomipramine  Paroxetine  Venlafaxine  Duloxetine  Escitalopram  Imipramine  Sertraline  Citalopram  Fluoxetine | Amitriptyline  Mirtazapine  Clomipramine  Venlafaxine  Imipramine  Paroxetine  Escitalopram  Duloxetine  Citalopram  Sertraline  Fluoxetine | Amitriptyline  Mirtazapine  Clomipramine  Venlafaxine  Imipramine  Duloxetine  Paroxetine  Escitalopram  Citalopram  Sertraline  Fluoxetine | Amitriptyline  Mirtazapine  Venlafaxine  Duloxetine  Paroxetine  Imipramine  Escitalopram  Sertraline  Fluoxetine  Citalopram |
| 4. As above, AND no worse than the best treatment^3^ | Amitriptyline  Clomipramine  Venlafaxine  Paroxetine  Escitalopram  Imipramine  Duloxetine  Sertraline  Citalopram | Amitriptyline  Clomipramine  Paroxetine  Venlafaxine  Duloxetine  Escitalopram  Imipramine  Sertraline  Citalopram | Mirtazapine  Clomipramine  Venlafaxine  Imipramine  Paroxetine  Escitalopram  Duloxetine  Citalopram | Mirtazapine  Clomipramine  Venlafaxine  Imipramine  Duloxetine  Paroxetine  Escitalopram  Citalopram | Mirtazapine  Venlafaxine  Duloxetine  Paroxetine  Imipramine  Escitalopram |
| 1. GRADE^2^ with threshold at zero. Treatments which are superior to placebo by 1 HAMD-17^2^ and inferior to no other treatment. (Number of treatments satisfying criterion 3 that they were superior to) | Amitriptyline (2)  Mirtazapine (2) | Amitriptyline (2)  Mirtazapine (2) | Amitriptyline (3)  Mirtazapine (2)  Venlafaxine (2) | Amitriptyline (3)  Mirtazapine (2)  Venlafaxine (1) | Amitriptyline (4)  Mirtazapine (2)  Venlafaxine (2) |

1 X better than Y means that the 95%CrI on the (X-Y) difference did not include zero

2 X better than Y by more than 1 HAMD-17 unit means that the 95% CrI on the (X-Y) difference did not include -1.0

3 X no worse than Y means that the 95%CrI on the (X-Y) difference did not include zero

# Appendix 4: Sensitivity analysis on the impact of ρ, the correlation between baseline and follow-up measures.

Calculation of the standard error (SE) for change-from-baseline requires a value for the correlation between baseline and follow-up measures (ρ, rho in the code). In our main analysis, we used a conservative value of 0.3 to reflect that these are reported measures and to avoid making the assumption of a strong positive correlation. Within a scenario analysis, this value was increased to 0.5 to assess the impact of assuming a stronger – though still conservative (Balk 2012) – correlation between baseline and follow-up measures.

The results of sensitivity analyses appear in Tables S6 and S7. Model fit statistics for RoM were largely unchanged with the increase in correlation from 0.3 to 0.5 (Table S6). Model fit – as captured by posterior mean residual deviance (D-bar) – was effectively unchanged for all SMD models. However, there were notable differences in pD with the increase in correlation: the number of effective parameters (pD) was approximately ten units higher in all SMD models. This higher pD results in a larger disparity in DIC and shrinkage between RoM and the SMD models when we assume a stronger correlation between baseline and follow-up measures.

Treatment effects were very similar between models assuming correlation at 0.3 and 0.5 (Figure S2). The sensitivity analysis gave very similar results for treatment recommendations compared to the main analysis, with the slight differences between models observed in the main analysis also seen in the sensitivity analysis (Table S7). In both analyses it appears that there was better discrimination between active treatments under the RoM model. This was evidenced by the smaller number of treatments that were not worse than the best treatment (decision rule 4), and the larger number of treatments to which the best treatment was superior (decision rule 5). There were small changes in decision rule 5 for Study-SMD models, with amitriptyline now estimated to be better than three treatments in the model without meta-regression, and venlafaxine now better than two treatments in the model with meta-regression.


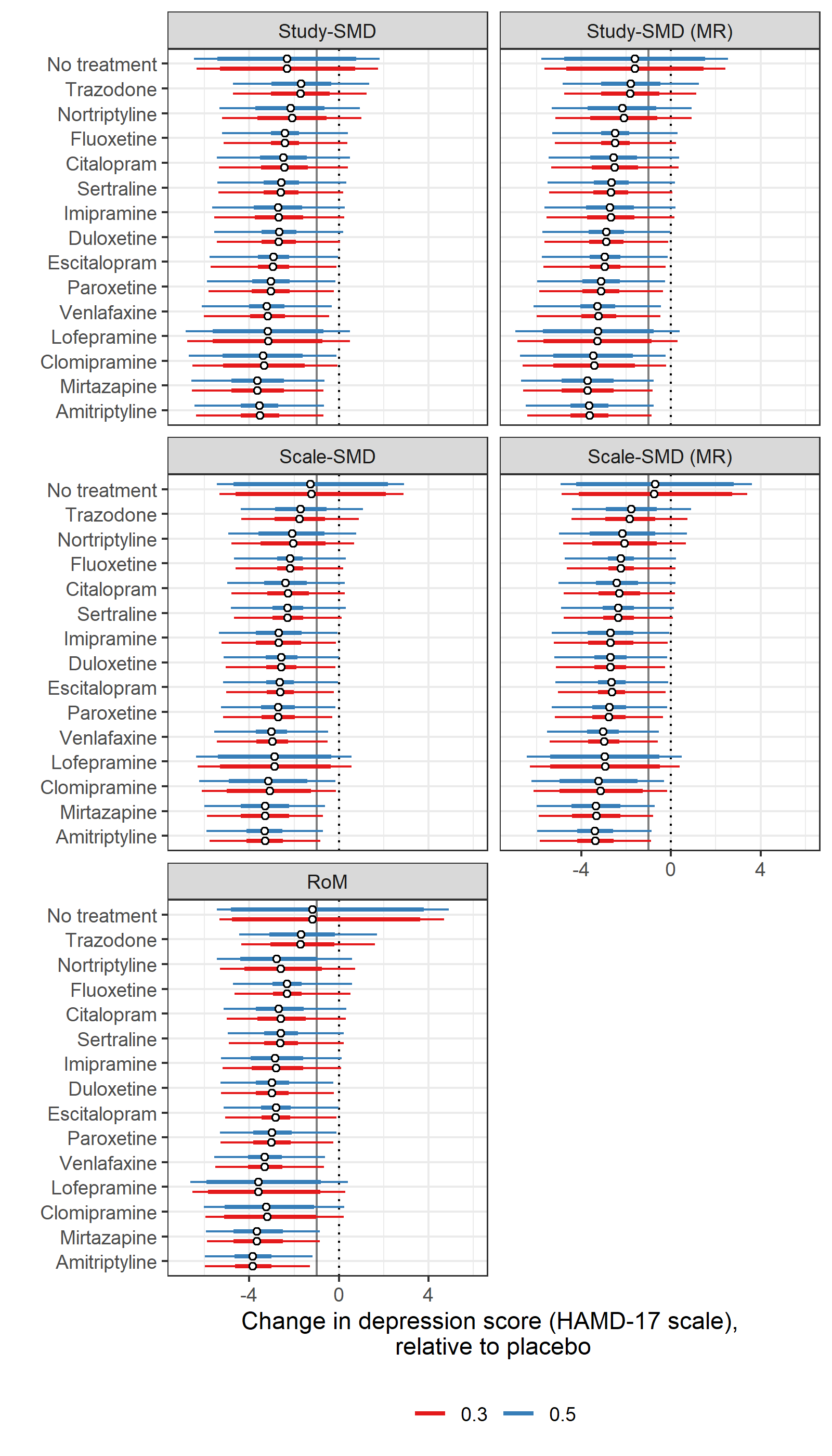


*Figure S2. Comparison of treatment effect estimates from base-case (correlation=0.3) and scenario analyses (correlation=0.5) for each NMA model. Points indicate median treatment effect, with the 95% credible interval shown with the thicker and the 95% predictive interval shown with the thinner line.*

**Table S6.** Model fit statistics for analyses where correlation between baseline and follow-up differed: total residual deviance (relative to 611 datapoints), DIC and shrinkage. Posterior median of between-studies SD with the 95% credible interval (CrI) on both the linear predictor scale and, for SMD models, on the HAMD-17 scale.

1. **Main analysis: correlation = 0.3**

| Model | Dbar | pD | DIC | % shrinkage | Regression  Coefficient  mean (95% CrI) | Between-study SD  Scale: linear predictor  Median (95% CrI) | Between-study SD  Scale: HAMD-17  Median (95% CrI) |
| --- | --- | --- | --- | --- | --- | --- | --- |
| Study SMD | 625.3 | 538.2 | 1163.6 | 16.73 | - | 0.34 (0.27, 0.41) | 1.35 (1.10, 1.65) |
| Study SMD (MR) | 625.3 | 537.5 | 1162.8 | 16.94 | -0.168  (-0.333, -0.002) | 0.33 (0.27, 0.40) | 1.33 (1.08, 1.62) |
| Scale SMD | 630.6 | 531.7 | 1162.3 | 18.23 | - | 0.29 (0.23, 0.36) | 1.18 (0.93, 1.45) |
| Scale SMD (MR) | 631.3 | 531.7 | 1163.0 | 18.27 | -0.121  (-0.272, 0.030) | 0.29 (0.23, 0.36) | 1.17 (0.92, 1.44) |
| Ratio of Means | 629.0 | 521.9 | 1150.9 | 20.49 | - | 0.09 (0.07, 0.12) | *Not calculable* |

1. **Sensitivity analysis: correlation = 0.5**

| Model | Dbar | pD | DIC | % shrinkage | Regression  Coefficient  mean (95% CrI) | Between-study SD  Scale: linear predictor  Median (95% CrI) | Between-study SD  Scale: HAMD-17  Median (95% CrI) |
| --- | --- | --- | --- | --- | --- | --- | --- |
| Study SMD | 626.6 | 546.6 | 1173.2 | 14.81 | - | 0.35 (0.28, 0.42) | 1.39 (1.14, 1.69) |
| Study SMD (MR) | 626.2 | 546.2 | 1172.4 | 14.92 | -0.167  (-0.333, -0.003) | 0.34 (0.28, 0.41) | 1.38 (1.12, 1.67) |
| Scale SMD | 631.1 | 542.6 | 1173.7 | 15.72 | - | 0.31 (0.25, 0.38) | 1.23 (1.00, 1.51) |
| Scale SMD (MR) | 631.2 | 542.3 | 1173.4 | 15.84 | -0.117  (-0.268, 0.037) | 0.31 (0.24, 0.37) | 1.22 (0.98, 1.50) |
| Ratio of Means | 627.1 | 521.4 | 1148.5 | 20.59 | - | 0.10 (0.07, 0.12) | *Not calculable* |

**Table S7.** Treatment recommendations on each model from five decision rules. Bold text indicates differences noted in the scenario analysis where correlation was set to 0.5.

| Decision rule | Study SMD | Study SMD  Meta-regression | Scale-SMD | Scale-SMD  Meta-regression | RoM |
| --- | --- | --- | --- | --- | --- |
| 1. Single best treatment | Mirtazapine | | Amitriptyline | | |
| 1. Treatments better than placebo^1^ | All active treatments | | | | |
| 1. Treatments better than placebo by 1 HAMD-17^2^ | Mirtazapine  Amitriptyline  Clomipramine  Venlafaxine  Paroxetine  Escitalopram  Imipramine  Duloxetine  Sertraline  Citalopram  Fluoxetine | Mirtazapine  Amitriptyline  Clomipramine  Venlafaxine  Paroxetine  Escitalopram  Duloxetine  Imipramine  Sertraline  Citalopram  Fluoxetine | Amitriptyline  Mirtazapine  Clomipramine  Venlafaxine  Paroxetine  Imipramine  Escitalopram  Duloxetine  Sertraline  Citalopram  Fluoxetine | Amitriptyline  Mirtazapine  Clomipramine  Venlafaxine  Paroxetine  Duloxetine  Imipramine  Escitalopram  Sertraline  Citalopram  Fluoxetine | Amitriptyline  Mirtazapine  Venlafaxine  Clomipramine  Paroxetine  Duloxetine  Escitalopram  Imipramine  Sertraline  Citalopram  Fluoxetine |
| 1. Treatments better than placebo by 1 HAMD-17^2^, AND no worse than the best treatment^3^ | Amitriptyline  Clomipramine  Venlafaxine  Paroxetine  Escitalopram  Imipramine  Duloxetine  Sertraline  Citalopram | Amitriptyline  Clomipramine  Venlafaxine  Paroxetine  Escitalopram  Duloxetine  Imipramine  Sertraline  Citalopram | Mirtazapine  Clomipramine  Venlafaxine  Imipramine  Paroxetine  Escitalopram  Duloxetine  Citalopram | Mirtazapine  Clomipramine  Venlafaxine  Imipramine  Duloxetine  Paroxetine  Escitalopram  Citalopram | Mirtazapine  Venlafaxine  Clomipramine  Duloxetine  Paroxetine  Imipramine  Citalopram |
| 1. GRADE, with threshold at zero.   Treatments which are superior to placebo by 1 HAMD-17^2^ and inferior to no other treatment. (Number of treatments satisfying criterion (3) that they were superior to) | **Amitriptyline (2) +1**  Mirtazapine (2)  Venlafaxine (2) | Amitriptyline (3)  Mirtazapine (2)  **Venlafaxine (1) +1** | Amitriptyline (3)  Mirtazapine (2)  Venlafaxine (2) | Amitriptyline (3)  Mirtazapine (2)  Venlafaxine (2) | Amitriptyline (4)  Mirtazapine (2)  Venlafaxine (2) |

^1^ X better than Y means that the 95%CrI on the (X-Y) difference did not include zero

^2^ X better than Y by more than 1 HAMD-17 unit means that the 95% CrI on the (X-Y) difference did not include -1.0

^3^ X no worse than Y means that the 95%CrI on the (X-Y) difference did not include zero

**REFERENCES**

1. Balk EM, Earley A, Patel K, Trikalinos TA, Dahabreh IJ. Empirical assessment of within-arm correlation imputation in trials of continuous outcomes. Rockville: Agency for Healthcare Research and Quality; 2012.
2. Brignardello-Petersen R, Florez ID, Izcovich A, et al. GRADE approach to drawing conclusions from a network meta-analysis using a minimally contextualised framework. *BMJ* 2020; **371**: m3900.
3. Daly C, Welton, S.J., Dias, S., Anwer,S., Ades, A.E. Meta-Analysis of Continuous Outcomes. Guideline Methodology Document 2: NICE Guidelines Technical Support Unit 2021.
